# Measuring Knowledge of Community Health Workers at the Last Mile in Liberia: Feasibility and Results of Clinical Vignette Assessments

**DOI:** 10.9745/GHSP-D-20-00380

**Published:** 2021-03-15

**Authors:** Jordan Downey, Anne H. McKenna, Savior Flomo Mendin, Ami Waters, Nelson Dunbar, Lekilay G. Tehmeh, Emily E. White, Mark J. Siedner, Raj Panjabi, John D. Kraemer, Avi Kenny, E. John Ly, Jennifer Bass, Kuang-Ning Huang, M. Shoaib Khan, Nathan Uchtmann, Anup Agarwal, Lisa R. Hirschhorn

**Affiliations:** a Last Mile Health, Boston, MA, USA.; b University of Texas Southwestern, Division of Combined Medicine and Pediatrics, Dallas, TX, USA.; c Liberia Ministry of Health & Social Welfare, Monrovia, Liberia.; d Massachusetts General Hospital, Harvard Medical School, Boston, MA, USA.; e Brigham & Women's Hospital, Harvard Medical School, Boston, MA, USA.; f Georgetown University, Department of Health Systems Administration, Washington, DC, USA.; g University of Washington, Department of Biostatistics, Seattle, WA, USA.; h University of Washington, Department of Family Medicine, Seattle, WA, USA.; i Northwestern University Feinberg School of Medicine, Chicago, IL, USA.

## Abstract

**Introduction:**

Community health workers (CHWs) can provide lifesaving treatment for children in remote areas, but high-quality care is essential for effective delivery. Measuring the quality of community-based care in remote areas is logistically challenging. Clinical vignettes have been validated in facility settings as a proxy for competency. We assessed feasibility and effectiveness of clinical vignettes to measure CHW knowledge of integrated community case management (iCCM) in Liberia's national CHW program.

**Methods:**

We developed 3 vignettes to measure knowledge of iCCM illnesses (malaria, diarrhea, and pneumonia) in 4 main areas: assessment, diagnosis, treatment, and caregiver instructions. Trained nurse supervisors administered the vignettes to CHWs in 3 counties in rural Liberia as part of routine program supervision between January and May 2019, collected data on CHW knowledge using a standardized checklist tool, and provided feedback and coaching to CHWs in real time after vignette administration. Proportions of vignettes correctly managed, including illness classification, treatment, and referral where necessary, were calculated. We assessed feasibility, defined as the ability of clinical supervisors to administer the vignettes integrated into their routine activities once per year for each CHW, and effectiveness, defined as the ability of the vignettes to measure the primary outcomes of CHW knowledge of diagnosis and treatment including referrals.

**Results:**

We were able to integrate this assessment into routine supervision, facilitate real-time coaching, and collect data on iCCM knowledge among 155 CHWs through delivery of 465 vignettes. Diagnosis including severity was correct in 65%–82% of vignettes. CHWs correctly identified danger signs in 44%–50% of vignettes, correctly proposed referral to the facility in 63% of vignettes including danger signs, and chose correct lifesaving treatment in 23%–65% of vignettes. Both diagnosis and lifesaving treatment rates were highest for malaria and lowest for severe pneumonia.

**Conclusion:**

Administration of vignettes to assess knowledge of correct iCCM case management was feasible and effective in producing results in this setting. Proportions of correct diagnosis and lifesaving treatment varied, with high proportions for uncomplicated disease, but lower for more severe cases, with accurate recognition of danger signs posing a challenge. Future work includes validation of vignettes for use with CHWs through direct observation, strengthening supportive supervision, and program interventions to address identified knowledge gaps.

## Abstract (precis)

We integrated clinical vignettes into routine programmatic supervision to assess community health worker knowledge of integrated community case management in rural Liberia. Results included higher rates of correct diagnosis and lifesaving treatment for uncomplicated disease than for more severe cases, with accurate recognition of danger signs posing a challenge.

## Teaser

**Key Findings:**

- Administration of vignettes in a community setting in rural Liberia was feasible and effective for measuring community health worker (CHW) knowledge of case management for the 3 major causes of child mortality addressed by integrated management of childhood illness.
- Correct diagnosis and lifesaving treatment for uncomplicated malaria were high, but correct diagnosis and treatment rates for complicated conditions including pneumonia with a danger sign were low.
- Although use of existing digital and paper-based clinical decision support job aids was inconsistent, CHWs who used them had statistically significant higher rates of correct diagnosis and treatment.

**Key Implications:**

- Improving the quality of CHW-delivered care is an important component of achieving universal health coverage, but gaps exist in how to measure knowledge and quality of delivery in remote settings.
- Clinical vignettes offer a potential approach for program implementers and policy makers to measure CHW knowledge, as a first step to incorporating assessments of the quality of CHW-delivered care into national CHW programs.

## INTRODUCTION

Over the last decade, community health workers (CHWs) have played a growing role in primary health care delivery in ensuring effective, accessible care in low-resource settings.^1^ Many countries have implemented integrated community case management (iCCM) programs to train CHWs to manage malaria, diarrhea, and respiratory illness in children under 5. Studies have suggested that iCCM increases treatment seeking, decreases morbidity, and may decrease mortality in children under 5.^1^^–^^3^ However, in many low- and middle-income countries (LMICs), quality of this care varies widely or is unknown,^2^^,^^4^ potentially limiting the impact of community-oriented primary health care programs. One recent review showed that health care providers, including CHWs, in 18 LMICs completed only 47% of recommended care items in evidence-based guidelines (determined through observations of consultations), with wide variation across countries.^5^ To address these quality of care challenges, the World Health Organization (WHO) and others have developed a combination of recommendations for optimizing CHW programs, including competency-based training, assessment, and certification, as well as supportive supervision.^6^

Despite these recommendations, there is no universally accepted or validated method to measure the quality of CHW-delivered care in the field. For facility-based health care providers, several assessment types have been widely used, including clinical vignettes, direct observation, standardized patients, chart reviews, patient surveys, and simulations.^7^^–^^9^ Techniques such as written tests and clinical vignettes assess provider knowledge, which has been shown to affect actual practice quality.^10^ However, there is some evidence that suggests provider knowledge assessment is more indicative of maximum possible performance than actual performance.^11^^,^^12^ Other techniques, such as direct observation of treatment, chart review, standardized patients, and interviews with caretakers can assess care delivery and quality directly. Some of these techniques can also capture components of experiential quality, including communication and respect, recognized as a critical quality domain of people-centered care.^13^ However, each assessment method has challenges in terms of cost, feasibility, and accuracy, particularly when applied to providers in remote settings. Chart or register review is inexpensive and feasible but can be limited by data quality. Although more direct assessments of CHW skills, such as direct observation in the field, are more reliable, they pose logistical challenges due to the remote locations and low caseloads of many CHW work environments, making it difficult to directly observe and assess quality of care. A more logistically feasible option is gathering geographically remote CHWs in a facility setting to directly observe provision of care, but removing CHWs from the community setting in which they routinely work may change how they provide care, result in gaps in care, and overestimate quality due to the Hawthorne effect.^7^^,^^8^^,^^12^

There is no universally accepted or validated method to measure the quality of CHW-delivered care in the field.

Vignettes are widely accepted as a measure of competency in most settings, and there is growing evidence of their validity as a proxy for measuring care delivery quality for facility-based health workers,^11^ as well as emerging evidence of their validity for measuring CHW-delivered care quality. In clinical vignettes, a trained assessor presents health care providers with an open-ended fictional clinical scenario of a sick patient who is brought for diagnosis and treatment and assesses their proposed management of the case measured based on national standards of care. A community-based study involving CHWs in Malawi showed that clinical vignettes estimated correct diagnosis and treatment to within 9 percentage points compared with direct observation and re-examination. This method offers an option for assessing CHW knowledge and potential insights into care delivery quality in their own communities even in remote rural settings where caseloads are small and access for observation is challenging.^7^ It also provides the opportunity for supportive supervision in real time based on CHW responses. However, the feasibility and utility of this method are relatively untested in remote rural CHW work environments.

A study of CHWs in Malawi showed that clinical vignettes estimated correct diagnosis and treatment to within 9 percentage points vs. direct observation and reexamination.

Last Mile Health (LMH) has partnered with the Liberia Ministry of Health since 2007 to provide primary care in remote communities in Liberia. In 2015, the Ministry of Health began to design a national community health program centered around a cadre of paid and supervised CHWs, known as community health assistants (CHAs), each of whom is recruited from the community he or she serves and supervised by a facility-based, clinically-trained Ministry of Health employee (primarily nurses).^14^ These Ministry of Health supervisors are called Community Health Services Supervisors (CHSSs). In Grand Bassa, Grand Gedeh, and Rivercess, counties where LMH directly supports national CHA program implementation, additional LMH clinician supervisors trained in the CHW curriculum, called quality assurance officers (QAOs), support the CHSSs by coaching them in supportive supervision designed to improve quality of care delivery.

The CHA program is currently being scaled up, with the goal of reaching the estimated 1.2 million Liberians who live farther than 5 kilometers from the nearest health facility. Although iCCM services are monitored routinely by CHSSs through review of CHW-maintained registers of care provided to patients, registers only capture the CHW's recorded diagnosis and treatment, posing potential data quality challenges as well as an inability to measure the quality of diagnosis and related treatment delivered.

We describe the feasibility and results of using clinical vignettes to measure CHW knowledge of community-based case management of sick children under 5 in remote rural Liberia integrated into existing supportive supervision. These vignettes focus on the main illnesses for which CHWs receive iCCM training in Liberia: malaria, respiratory infection, and diarrhea. This work was designed to facilitate programmatic quality improvement through supplementing routine supportive supervision for CHWs in LMH-supported counties. It was also designed to inform the Liberia National CHA Program about potential feasible and scalable approaches to measure quality of CHW-delivered care, guide supportive supervision to address gaps in knowledge, and inform future studies to validate vignettes as a proxy for care competency. The results are also relevant for similar programs focused on ensuring that care delivered by CHWs is of the highest quality regardless of setting.

This work aimed to inform the Liberia National CHA Program about potential feasible and scalable approaches to measure quality of CHW care and guide supportive supervision to address knowledge gaps.

## METHODS

### Liberia's National CHA Program

CHWs in Liberia are recruited from the communities they serve and are required to have at least a sixth-grade education level, although in some rural communities where this is not feasible, good candidates with less education may be hired. They are paid, regularly supervised by clinicians (CHSSs), and trained in 4 modules for approximately 2 weeks per module before initiating field activities. The 4 modules include: (1) registration and community-based disease surveillance; (2) reproductive, maternal, and neonatal health; (3) child health, including iCCM of common childhood illnesses; and (4) additional services including management of HIV, TB, leprosy, mental health, and first aid. CHWs are also equipped with decision support job aids to reinforce their training and help them provide services within each module. These include printed job aids, sick child data collection registers, and mobile-phone-based data collection and clinical decision support tools. Job aids include protocols for assessment, classification, and treatment; visuals to aid in symptom and medication recognition; guidance for providing health promotion education to community members; and tips and reminders for advising caregivers.^15^ CHWs who complete module 3 in child health are expected to diagnose and treat uncomplicated malaria, diarrhea, and pneumonia as defined by national protocols and to recognize and refer moderate and severe malnutrition. They are also expected to recognize danger signs that require urgent referral to a health facility. Job aids for module 3 focus on helping CHWs correctly diagnose and treat iCCM illnesses, including recognizing danger signs and appropriate referral. Although job aids are provided during training and CHW supervisors encourage their use, CHWs are not required to use them during consultations with patients.

### Study Population and Setting

The study was completed in 3 counties (Rivercess, Grand Gedeh, and Grand Bassa) supported by LMH. QAOs are supposed to visit each CHW in their catchment area at least once per year as part of their routine supervision responsibilities. Because the information presented here was collected as part of this routine supervision, CHWs were not selected randomly but were included in this assessment if they were actively working in 1 of these counties and received a QAO visit between January 2019 and May 2019.

### Vignette Development & QAO Training

Vignettes were developed for the 3 main illnesses treated through iCCM (malaria, diarrhea, and pneumonia) based on the WHO Health Facility Survey observation tools (used to evaluate the quality of care delivered to sick children attending outpatient facilities),^16^ and the national policy and job aids developed for the Government of Liberia's National CHA Program. Vignettes captured knowledge in 4 main areas: assessment, diagnosis, treatment, and caregiver instructions, all of which are areas targeted by QAO coaching of the CHW in the field.

We piloted the approach with an initial set of 4 vignettes with 97 CHWs in Rivercess and Grand Gedeh counties. The initial vignettes included uncomplicated malaria, acute respiratory illness with a danger sign (chest in-drawing), and uncomplicated diarrhea. The pilot was carried out by QAOs trained to administer the vignettes in their respective counties. We identified several challenges, including the length of time it took to conduct the assessment with 4 vignettes, poor quality of videos used to prompt CHW assessment of respiratory distress, and confusing design of forms used to collect data, especially on dosing selection. In response to these challenges, we revised the vignettes, reducing the number of vignettes from 4 to 3 and modifying response forms to make data collection requirements clearer to more accurately collect dosing information. Although resource constraints did not allow for creation of better quality videos, we removed the necessity of using videos for identifying danger signs by modifying the pneumonia vignette such that the danger sign was the length of time the child had a cough as opposed to the respiratory rate (although the video was still necessary for determining respiratory rate to correctly diagnose pneumonia). We also modified the initial vignettes (which included uncomplicated malaria, acute respiratory illness with a danger sign of chest in-drawing, and uncomplicated diarrhea) to address different sick child scenarios with children of different ages and illnesses. This ensured no skewing of the results by CHWs who had already completed the assessment with the original vignettes and received feedback on their performance.

In response to challenges identified in the pilot effort, we reduced the number of vignettes and revised response forms to make data collection requirements clearer.

The new vignettes included acute respiratory illness with a danger sign (cough for more than 14 days), diarrhea with a danger sign (mid-upper arm circumference (MUAC) in the red region), and uncomplicated malaria (Supplement 1). Two vignettes were designed to include danger signs due to CHW difficulty with recognizing danger signs identified through the pilot. Vignettes were developed through an iterative process of drafting, review by field-based staff, and revision, and were translated into Liberian English before use.

QAO training was developed based on the WHO Health Facility Survey.^16^ QAOs were trained on how to explain and administer the vignette, including instructing the CHW to describe or show what they would do if a real child was present, reminding the CHW to use any tools they normally use in their everyday work and role-playing the part of a caretaker. They were also trained to record the CHW's diagnosis and treatment for each vignette using a standardized data collection tool; to mark whether the assessment, diagnosis, treatment and caregiver advice were correct; and to note which job aids the CHW used, if any.

### Vignette Administration

QAOs administered each of the 3 open-ended vignettes to individual CHWs in the CHW community during their routine supervision visits. The vignettes were presented through playing the role of a sick child's caregiver, and responses were manually recorded using a standardized data collection checklist tool. Vignettes and data collection tools were designed to collect data on CHW knowledge of assessment, diagnosis, treatment and caregiver instructions, as well as CHW use of job aids, primarily to facilitate QAO feedback to and coaching of the CHW in the field. Our primary outcomes were CHW knowledge of correct diagnosis and treatment including referral if necessary for each vignette. Secondary outcomes included CHW knowledge of assessment tasks and caregiver instructions and CHW use of job aids. CHWs were instructed before the start of the vignette to use any materials they would use in their normal practice that are components of the national program. If CHWs asked about respiratory rate or chest in-drawing, they were shown a video of a child with the respiratory status of the case to evaluate for presence of respiratory distress. If the CHW wanted to perform a rapid diagnostic test (RDT) for malaria, they were asked to explain the steps involved and demonstrate everything except the finger prick on the QAO, and QAOs provided the results appropriate for each vignette (positive for the malaria vignette, negative for the others). CHWs also had the opportunity to demonstrate their ability to correctly perform MUAC measurement to evaluate children for malnutrition, a screening procedure that they are supposed to perform during every consultation regardless of symptoms. CHWs were asked to describe the steps of the procedure and demonstrate the measurement on the QAO or another volunteer. After performing the assessment, CHWs were expected to make a diagnosis and recommend the correct medication and management of the case as it would be communicated to an actual caregiver. CHWs were also expected to refer the child to a health facility if danger signs were present; this was considered a part of correct diagnosis and treatment. After completion of all the assessments, QAOs gave CHWs feedback on their performance and coached them in improving their skills in areas that were challenging for them. All data were collected on paper forms and entered into a custom MySQL database by LMH staff.

### Data Analysis

We defined feasibility as ability of QAOs to administer the vignettes integrated into their routine activities once per year for each CHW (in other words, ability to assess half of all CHWs in their catchment areas over a 6-month period), in counties directly supported by LMH.

Effectiveness was defined as the ability of the vignettes to measure the primary outcomes of CHW knowledge of diagnosis and treatment including referrals. Three indicators were used to assess knowledge for each vignette: (1) correct diagnosis, defined as assigning the correct clinical diagnosis and presence or absence of the correct danger sign; (2) correct lifesaving treatment, defined as providing lifesaving treatment including describing the correct dose for amoxicillin, artemisinin-combined treatment, and any oral rehydration solution and recommending referral if a danger sign was present; and (3) fully correct treatment, including providing correct dosing specifications for all medications including paracetamol for malaria and oral rehydration solution for diarrhea (Table 1). Correct dosing was defined based on Liberia's national CHW policy^17^ including dose, duration, and frequency. For the vignette involving diarrhea, zinc was not included in the definition of lifesaving or fully correct treatment because Liberian national guidelines recommend immediate referral without giving zinc when a child is diagnosed with diarrhea and a danger sign.

**TABLE 1.** Clinical Vignettes and Indicators Used to Assess CHW Knowledge of Community-Based Management of Sick Children Under Age 5 in 3 Rural Counties, Liberia

Vignette	Case Description	Indicators		
		Correct Diagnosis	Correct Lifesaving Treatment	Fully Correct Treatment
**Pneumonia with danger sign**	Male, aged 4 years, presents with cough for 2 weeks	Pneumonia + danger sign (cough 2+ weeks)	Refer to facility; amoxicillin given (must be correct dose)	Refer to facility; amoxicillin given (must be correct dose)
**Diarrhea with danger sign**	Female, aged 8 months, presents with running stomach and malnutrition	Diarrhea + danger sign (red MUAC)	Refer to facility; ORS given regardless of dose	Refer to facility; ORS given (must be correct dose)
**Uncomplicated malaria**	Female, aged 2 years, presents with weakness and fever	Malaria	ACT given (must be correct dose)	ACT given (must be correct dose); paracetamol given (must be correct dose)

Abbreviations: ACT, artemisinin-combined treatment; CHW, community health worker; MUAC, mid-upper arm circumference; ORS, oral rehydration solution.

Effectiveness was defined as the vignettes' ability to measure the primary outcomes of CHW knowledge of diagnosis and treatment including referrals.

Proportions of correct diagnosis or treatment and 95% confidence intervals (CIs) were calculated using the definitions in Table 1.

We also collected and analyzed data on several other aspects of the case management process, including assessment (gathering information on the sick child and performing diagnostic tests), use of clinical decision support job aids, and provision of anticipatory guidance and health promotion messages to caregivers. CHW clinical support job aids and training handbooks remind the user that according to national policy, CHWs should ask 8–9 information gathering questions and perform 1–5 diagnostic tests, depending on the presenting case.

We collected these data to facilitate QAO coaching and feedback in the field as part of the assessment process and to better understand common pitfalls that may lead to suboptimal case management. However, we chose not to include these tasks in definitions for correct diagnosis and treatment; the emphasis was placed on whether actual diagnosis recorded and treatment chosen was correct and not on the process followed to inform the outcomes.

The proportion of CHWs performing each potential information gathering and diagnostic test, as well as the proportion who performed each diagnostic test correctly, were calculated with 95% CIs. The proportion of CHWs appropriately advising caregivers was also calculated for each vignette and across vignettes, with 95% CIs. To understand the extent to which gaps in knowledge might be filled through better use of existing tools, we also examined the proportion of correct diagnosis and treatment decisions among those who used or did not use job aids (Supplement 2). All analysis was carried out using Stata version 14 (StataCorp).

### Ethical Considerations

Data collection tools and vignettes were reviewed and approved by institutional review boards in both the United States and Liberia as part of a routine programmatic institutional review board submission, so informed consent was not required by the review boards.

## RESULTS

### Vignette Administration

Between January 1, 2019 and May 15, 2019, 155 CHWs were evaluated using all 3 vignettes (total 465 vignettes): 40 (26%) in Grand Bassa County, 37 (24%) in Grand Gedeh County, and 78 (50%) in Rivercess County. As planned, QAOs were able to administer all 3 vignettes to half of all active CHWs in their respective catchment areas in a 6-month period, reflecting feasibility of assessing all CHWs once per year and integrating vignettes into routine supervision in counties managed by LMH.

Each evaluation session took approximately 2 hours to administer the 3 vignettes.

### Vignette Results

The percentage of CHWs correctly diagnosing each illness are displayed in the Figure. More than 50% of CHWs (range 57%–82% based on case) determined the primary diagnosis correctly. However, CHWs correctly diagnosed the illness and severity of illness (danger signs) in only 26% of pneumonia vignettes (95% CI=20%, 34%) and only 34% of diarrhea vignettes (95% CI=27%, 42%) (Figure). The most common error was missing the presence of a danger sign or incorrectly identifying the presence of a danger sign. CHWs correctly diagnosed the danger sign in 50% (95% CI=42%, 58%) of the pneumonia vignettes and 44% (95% CI=36%, 52%) of the diarrhea vignettes.

**FIGURE:**
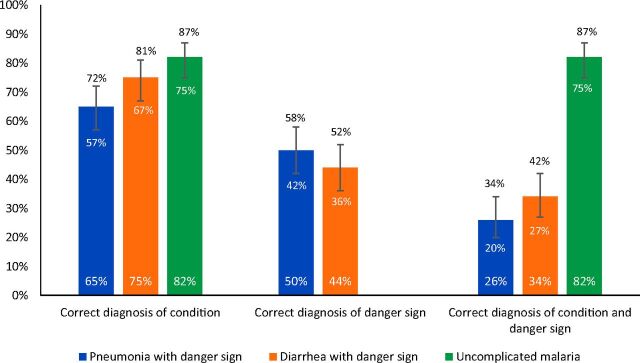
Correct Diagnoses Provided by CHWs During Clinical Vignettes to Assess Case Management of Sick Children Under 5 in 3 Rural Counties, Liberia

Correct treatment rates are presented in Table 2. CHWs prescribed correct lifesaving treatment in 23% of pneumonia vignettes (95% CI=17%, 31%), 50% of diarrhea vignettes (95% CI=42%, 58%), and 65% of malaria vignettes (95% CI=57%, 72%) (Table 2). Fully correct treatment was also prescribed in 21% of diarrhea vignettes (95% CI=15%, 29%) and 28% of malaria vignettes (95% CI=21%, 35%). Since the definitions of correct lifesaving and fully correct treatment were the same for the pneumonia vignette, CHWs prescribed fully correct treatment in the same percentage of pneumonia vignettes (23%, 95% CI=17%, 31%). In the 2 cases with danger signs, CHWs proposed referral to the facility 63% of the time for both vignettes (95% CI=55%, 70%). CHWs incorrectly identified a danger sign when there was no danger sign in 13% (95% CI=9%, 20%) of malaria vignettes.

**TABLE 2.** Treatment Recommended by CHWs During Clinical Vignettes Assessing the Correct Management of Sick Children Under 5 in 3 Rural Counties, Liberia

Vignette and Correct Treatment Activity	Activity Type	Percentage of Vignettes in Which CHWs Recommended Activity, % (95% CI) (N=155)
Pneumonia with danger sign		
Treatment with amoxicillin	Lifesaving	62 (54, 69)
Correct amoxicillin dose	Lifesaving	39 (32, 47)
Referral to the facility	Lifesaving	63 (55, 70)
Overall correct lifesaving treatment	Lifesaving	23 (17, 31)
Overall fully correct treatment	Fully correct	23 (17, 31)
Diarrhea with danger sign		
Treatment with ORS	Lifesaving	78 (71, 84)
Correct ORS dose	Fully correct	46 (39, 54)
Referral to the facility	Lifesaving	63 (55, 70)
Overall correct lifesaving treatment	Lifesaving	50 (42, 58)
Overall fully correct treatment	Fully correct	21 (15, 29)
Uncomplicated malaria		
Treatment with ACT	Lifesaving	74 (67, 81)
Correct ACT dose	Lifesaving	65 (57, 72)
Treatment with paracetamol	Fully correct	73 (65, 79)
Correct paracetamol dose	Fully correct	39 (32, 47)
Overall correct lifesaving treatment	Lifesaving	65 (57, 72)
Overall fully correct treatment	Fully correct	28 (21, 35)

Abbreviations: ACT, artemisinin-combined treatment; CHW, community health worker; ORS, oral rehydration solution.

### CHW Case Management Process

#### Gathering Information and Diagnostic Tests

During the diarrhea vignette, 91% of CHWs (95% CI=85%, 95%) asked how long the child had diarrhea, 55% (95% CI=47%, 63%) asked how many loose stools per day, and 68% (95% CI=60%, 75%) asked if there was any blood in the stool. Only 14% of CHWs (95% CI=9%, 21%) checked for the danger sign of bilateral pitting edema indicating malnutrition. Of the 54% of CHWs (95% CI=46%, 61%) that checked the child's MUAC, 43% (95% CI=33%, 54%) demonstrated the skill correctly. Performing this diagnostic test was essential to diagnosing the danger sign of red MUAC.

During the pneumonia vignette, 88% of CHWs (95% CI=81%, 92%) asked how long the child had been coughing, 43% (95% CI=35%, 51%) said they would count the child's respiratory rate, and 26% (95% CI=20%, 34%) said they would check for chest in-drawing.

During the malaria vignette, 77% (95% CI=70%, 83%) asked if the child had a fever (the prompting question was “My child is weak,” which is a common presenting complaint for malaria), and 65% (95% CI=57%, 72%) asked how long the child had a fever. Although accurate malaria diagnosis was high, in 32% of malaria vignettes, the CHW did not state that they would perform an RDT (95% CI=25%, 39%). Of those who did perform an RDT, 75% (95% CI=66%, 83%) demonstrated the skill correctly.

#### Using Job Aids

Only 30% of CHWs (95% CI=23%, 37%) used job aids in all 3 cases, and average job aid use across vignettes was 43% (95% CI=38%, 47%). Correct diagnosis was 15–33 percentage points higher and correct lifesaving treatment was 12–30 percentage points higher for CHWs who used job aids versus those who did not use job aids. These differences were statistically significant for correct diagnosis and correct lifesaving treatment for all vignettes (*P* values ranged from <.001 for correct diagnosis in the diarrhea vignette to .0362 for correct lifesaving treatment in the pneumonia vignette).

CHWs used job aids in 43% of vignettes; for those that used job aids, correct diagnosis and correct lifesaving treatment was significantly higher compared to those who didn't use them.

#### Giving Advice to Caregivers

CHWs advised the caregiver on how to give treatment at home in 45% of vignettes (95% CI=40%, 50%) and discussed follow-up with the caregiver in 46% (95% CI=41%, 51%) of vignettes (Supplement 2 includes full details).

## DISCUSSION

We found that administration of vignettes in the field was feasible and effective for producing information on CHW knowledge of case management for the 3 main areas of iCCM diagnosis and treatment in their scope, in counties supported by LMH. The vignettes effectively identified a number of gaps in CHW knowledge of correct care provision for common childhood illnesses under the national CHA program in rural Liberia. Although correct diagnosis and lifesaving treatment for uncomplicated malaria was high, recognition of correct danger signs and the ability to verbally describe correct dosage (in the absence of identifying regimens by visual cues such as medication packaging or color) were areas where additional support is needed. One possible reason for these low results is insufficient use of provided national program job aids and digital clinical support tools designed to remind CHWs of correct dosages and management. CHWs used provided digital or paper-based job aids in only 43% of vignettes, and correct diagnosis and treatment proportions were higher among CHWs who used their nationally distributed job aids than among those who did not. This highlights an opportunity to improve care through existing resources by understanding and addressing barriers to job aid use.

The vignettes effectively identified gaps in CHW knowledge of correct care provision for common childhood illnesses.

Assessing CHW knowledge using clinical vignettes was feasible in this setting, as administered by QAOs in counties supported by LMH. Given these results, these vignettes have been incorporated into regular program monitoring for geographies where LMH is supporting implementation within these counties, representing an important step forward for measurement of CHW quality of care in remote settings where caseloads are small and resources are limited. While the assessments using clinical vignettes did prolong routine supervision, it was possible to integrate these assessments into routine QAO activities without significantly decreasing the frequency of regular supervision carried out and provided an opportunity for real time feedback and program improvement. For example, some of the errors in dosing were found to be related to recent transitions from syrup to pill form of paracetamol and amoxicillin resulting in confusion about how to describe the correct dose. Although this was a problem for only some CHWs, especially those who had been previously trained in dosing syrup forms of these medications, additional training was conducted for all CHWs to support correct dosing, and revised job aids were distributed. Correct MUAC measurement techniques were also challenging for many CHWs, and additional training was conducted to improve these skills. There were also technical challenges identified. The videos used to allow CHWs to evaluate respiratory rate and to determine the diagnosis of pneumonia were found to be poor quality, which may have contributed to the low rates of correct diagnosis in the pneumonia case. New higher-quality videos are in development for future use.

Our findings on CHW knowledge of iCCM case management are similar to findings by Cardemil et al., who evaluated quality of care through case scenarios in Malawi, similar to our clinical vignettes in structure and content.^7^ Correct treatment for severe illness as measured by case scenarios ranged from 37% for scenarios involving children presenting with fast breathing (versus 23% in our results) to 70% for scenarios involving children presenting with diarrhea (versus 50% in our results) and was 95% for uncomplicated fever (versus 65% for our results).^7^ Factors that may contribute to higher quality of sick child care by CHWs in Malawi include that CHWs in Malawi have more training on average and are required to have higher educational attainment (tenth grade or higher versus sixth grade) compared to Liberian CHWs.^4^

Our findings are also similar to a number of studies that evaluated CHW quality of care for iCCM illnesses in children under 5 using direct observation. For example, incorrect diagnosis of a danger sign was seen in a study by Kelly et al. in 1 district in Kenya from 1998 to 2001 using direct observation and reexamination, which found that the proportions of children receiving correct adequate treatment ranged from 35.5% to 57.8% for children with a severe classification,^18^ compared to 23%–50% in our assessment. In a study in Tanzania also using direct observation and reexamination, CHWs were found to correctly refer children to a facility for danger signs only 66% of the time^19^ compared to 63% in our cohort. A similar study in Uganda found that an antibiotic was correctly prescribed for pneumonia only 40% of the time, somewhat lower than our rate of 62%.^20^ However, the same study also found higher CHW performance (87%–97% correct) in assessing respiratory rate, RDT use, correct illness classification, and correct antimalarial prescription when CHW performance was compared to a gold standard pediatrician's assessment, classification, and prescription. This is higher than our correct diagnosis of malaria and pneumonia (82% and 65%, respectively) and our correct antimalarial prescription of 65%. Although there were similarities between these studies and our findings, recruitment and training of CHWs varied between studies and study country settings, which are important factors that may have had an influence on quality of care (Supplement 3 includes full details of reviewed studies).

CHW knowledge of diagnosis and treatment of severe pneumonia with the presence of a danger sign was lower than for other vignettes, reflecting significant challenges with managing childhood pneumonia. In addition to the evidence presented here that supports management of severe illness by CHWs as a common challenge, there is also evidence of meaningful gaps in quality of facility-based childhood pneumonia management in LMICs. An assessment comparing facility-based care and CHW-delivered care for iCCM diseases in rural Uganda found that only 54% of children treated for pneumonia by any provider were appropriately assessed or treated, and only 62% of episodes of any iCCM illness were appropriately treated by public health facilities.^21^ In an assessment of facility-based quality of care in rural Zambia, only 21% of children with pneumonia received an appropriate diagnosis, and less than half of those diagnosed received appropriate antibiotic treatment.^22^ These findings highlight the importance of measuring and improving quality of iCCM care by both facility and community-based providers, with a particular emphasis on severe pneumonia.

### Limitations

Our study has several limitations. Clinical vignettes in our setting may not accurately reflect quality of care CHWs are delivering. Vignettes may overestimate true rates of correct diagnosis and treatment. For example, the study by Cardemil et al. compared quality of care results obtained through case scenarios to those obtained through direct observation and reexamination and showed that estimates of correct treatment for uncomplicated fever and diarrhea as measured through case scenarios were 9 percentage points higher than estimates obtained through direct observation and reexamination. This gap was even larger for cases involving fast breathing and severe illness, which may indicate that vignettes are an appropriate tool for estimating CHW performance for uncomplicated illness but less appropriate for cases involving fast breathing and severe illness.^7^ Vignettes may also underestimate quality of care provided, as routine steps such as performing a MUAC screen and looking for other danger signs may be more likely forgotten in a vignette setting. Additionally, CHWs may know medications by sight (such as color-coded dosing for ACT or blister packs facilitating correct dosing) but may not be able to describe specific doses. We took steps to minimize error by designing open-ended vignettes, training QAOs on vignette administration including role-playing a caretaker, and ensuring that all relevant skills were assessed using simulation or other methods when feasible (malaria diagnosis through either RDT administration up to blood draw or detailed description of the skill and ability to correctly perform malnutrition screening through demonstration of MUAC measurement skills on the QAO).

Because the assessment is carried out as part of routine programmatic activities, CHWs are chosen for the assessment based on QAO work plans and are not randomly selected. This may mean our results on quality of care are not generalizable to other CHWs in LMH-supported counties or in Liberia more broadly. Because this was a programmatic quality improvement exercise, we did not collect information on CHW demographics, so it was not possible for us to comment on how factors like age, educational level, and length of training might lead to variations in CHW performance.

Furthermore, the assessment was completed by LMH employees (QAOs) as opposed to supervisors hired by the Ministry of Health because resource constraints did not allow for hiring and training of equivalent Ministry of Health supervisors as part of this assessment. Therefore, feasibility of implementation at national scale throughout Liberia was not assessed, although the results can inform a future assessment of this. This potentially limits the generalizability of the feasibility results, given that this cadre of supervisors is not currently available to other counties in Liberia and a similar cadre in other CHW programs in other LMICs may not be able to spend the required 2 hours for administration and follow-up. Finally, CHWs were not familiar with the use of clinical vignettes, and some errors may reflect lack of experience with the assessment method, although this was not noted as a challenge in the work by Cardemil et al.

Given the limited number of studies evaluating the quality of CHW-delivered care in a community setting, more research is needed globally on the feasibility and accuracy of methods used to measure this quality of care. Research on quality improvement mechanisms for CHW programs is also urgently needed to respond to these results. The resulting deeper understanding of the quality of CHW-delivered care, underlying challenges, and mechanisms for improvement can help implementers develop and sustain programs that provide more patient-centered care and lead to better health outcomes. Although there are limitations to using clinical vignettes to evaluate quality of CHW-delivered care, our results combined with the global evidence suggest that clinical vignettes can be used to assess CHW knowledge in many different country and disease contexts. They can be adapted to test knowledge of either major causes of morbidity and mortality, or for rare presentations that may be difficult to assess through direct observation. Vignettes should be designed to evaluate case management according to national guidelines, including the scope of the CHW's role and the specific protocols CHWs use, and updated when treatment protocols change.

## CONCLUSION

To improve programmatic quality, our next steps include continuous refinement of this method based on the limitations and lessons learned laid out above, including creation of higher-quality videos and ongoing training for QAOs in vignette administration and coaching. In addition, we are planning to validate this method as an accurate measure of quality of care delivery through a study comparing direct observation and vignettes. With Liberia's Ministry of Health, we are also exploring ways this assessment can be incorporated into broader programmatic supervision, as well as testing a smartphone-enabled, multimedia learning management system with iCCM-focused (e.g., malaria, malnutrition) training modules to enhance CHW competencies.^23^^,^^24^ The results will help inform Liberia's national program on optimal ways to measure and improve CHW competency moving forward and contribute to the global knowledge base for best practices in assessing quality of CHW care in remote or rural settings.

In conclusion, we believe that vignettes are a valuable tool for assessing CHW knowledge of iCCM case management and may serve as a proxy for competency when direct observation of care provision is not feasible to identify in real time gaps in knowledge, potential gaps in practice, and targets for in-the-field coaching. As CHW programs expand to provide primary care delivered by frontline workers in remote settings, contributing to achieving universal health care, feasible and integrated performance measurement to inform supportive supervision is critical to ensuring high quality care.

## Supplementary Material

20-00380-Downey-Supplement2.pdf

20-00380-Downey-Supplement1.pdf

20-00380-Downey-Supplement3.pdf

## Notes

**Peer Reviewed**
