# Supplementary material for: Measuring Knowledge of Community Health Workers at the Last Mile in Liberia: Feasibility and Results of Clinical Vignette Assessments
Source: Glob Health Sci Pract. 2021 Mar 15;9(Suppl 1):S111–21. doi: 10.9745/GHSP-D-20-00380 (PMC7971375; doi:10.9745/GHSP-D-20-00380)
Supplement: 20-00380-Downey-Supplement2.pdf [file 20-00380-Downey-Supplement2.pdf]

Supplement to: Downey J, McKenna AH, Mendin SF, et al. Measuring knowledge of community health workers at the last mile in Liberia: feasibility and results of clinical vignette assessments. *Glob Health Sci Pract*. 2021;9(suppl 1). https://doi.org/10.9745/GHSP-D-20-00380

Supplement 2

Supplementary Table. Information gathering

Question	% of CHWs asking question during pneumonia vignette (95% CI)	% of CHWs asking question during diarrhea vignette (95% CI)	% of CHWs asking question during malaria vignette (95% CI)
Has the child had fever?	58 (50-66%)	70 (63-77%)	77 (70-83%)
Has the child had a cough?	N/A (presenting complaint)	64 (56-71%)	67 (59-74%)
Has the child had diarrhea?	57 (49-65%)	N/A (presenting complaint)	69 (61-76%)
Can the child drink or breastfeed?	26 (20-34%)	34 (26-41%)	45 (37-52%)
Has the child been vomiting everything?	30 (23-37%)	35 (28-43%)	35 (28-43%)
Has the child had any convulsions?	19 (14-26%)	24 (18-31%)	28 (22-36%)
Has the child already taken medication for this problem?	8 (4-13%)	12 (8-19%)	11 (7-17%)
Cough/fast breathing-specific questions:			
How long has the child been coughing?	88 (81-92%)	N/A	
Diarrhea-specific questions:			
How long has the child had diarrhea?	N/A	91 (85-95%)	N/A
How many loose stools per day?		55 (47-63%)	
Any blood in the stool?		68 (60-75%)	
Fever-specific questions:			
How long has the child had fever?	N/A		65 (57-72%)

The number and type of information gathering questions asked varied by vignette. Across all cases, most CHWs asked questions about the three most common presenting complaints for iCCM illnesses in children under five (58-77% of CHWs asked if the child had a fever, while 64-67% asked if the child had a cough and 57-69% asked if the child had diarrhea). During the pneumonia vignette, most (88%, 95% CI 81-92%) CHWs asked how long the child had been coughing (cough was the presenting complaint). During the diarrhea vignette, only 55% (95% CI

Supplement to: Downey J, McKenna AH, Mendin SF, et al. Measuring knowledge of community health workers at the last mile in Liberia: feasibility and results of clinical vignette assessments. *Glob Health Sci Pract*. 2021;9(suppl 1). https://doi.org/10.9745/GHSP-D-20-00380

47-63%) of CHWs asked how many loose stools the child was having per day, but most (68%, 95% CI 60-75%) asked if there was blood in the stool and how long the child had had diarrhea (91%, 95% CI 85-95%). During the malaria vignette, in which the presenting complaint was weakness, most CHWs asked if the child had a fever (77%, 95% CI 70-83%) and 65% (95% CI 57-72%) asked about the duration of the fever.

Supplementary Table 2: Assessment task and diagnostic test performance

Assessment task or diagnostic test	% of CHWs stating they would perform task during pneumonia vignette (95% CI)	% of CHWs stating they would perform task during diarrhea vignette (95% CI)	% of CHWs stating they would perform task during malaria vignette (95% CI)
Assess how the child looks	8 (4-13%)	10 (6-16%)	14 (9-21%)
Assess if the child's skin is hot to the touch	9 (5-15%)	10 (6-16%)	18 (13-25%)
Assess if the child is very sleepy or unconscious	10 (6-16%)	17 (12-24%)	21 (15-29%)
Check for bilateral pitting edema	10 (6-16%)	14 (9-21%)	15 (11-22%)
Check for chest in-drawing	26 (20-34%)	11 (7-17%)	6 (3-12%)
Of those who indicated they would perform the task, % demonstrating skill correctly	59 (42-73%)	47 (24-72%)	70 (31-92%)
Count respiratory rate	43 (35-51%)	10 (6-16%)	5 (2-9%)
Of those who indicated they would perform the task, % demonstrating skill correctly	39 (28-52%)	44 (20-70%)	86 (26-99%)
Check the child's MUAC	28 (21-35%)	54 (46-61%)	30 (24-38%)
Of those who indicated they would perform the task, % demonstrating skill correctly	65 (49-78%)	43 (33-54%)	57 (43-71%)
Malaria rapid diagnostic test	17 (12-24%)	18 (13-25%)	68 (61-75%)
Of those who indicated they	59 (39-77%)	39 (22-59%)	75 (66-83%)

Supplement to: Downey J, McKenna AH, Mendin SF, et al. Measuring knowledge of community health workers at the last mile in Liberia: feasibility and results of clinical vignette assessments. *Glob Health Sci Pract*. 2021;9(suppl 1). https://doi.org/10.9745/GHSP-D-20-00380

would perform the task, % demonstrating skill correctly			

During the pneumonia vignette, only about a quarter (26%, 95% CI 20-34%) of CHWs indicated they would check for chest in-drawing, and less than half (43%, 95% CI 35-51%) indicated they would count the respiratory rate. For each of these tasks, respectively 59% (95% CI 42-73%) and 39% (95% CI 28-52%) demonstrated the skill correctly using the provided video. It is worth noting that the poor quality of the videos likely contributed to these suboptimal scores. During the diarrhea vignette, 54% (95% CI 46-61%) of CHWs indicated they would perform a mid-upper arm circumference (MUAC) assessment, a task necessary to correctly diagnose the danger sign in this case (red MUAC). During the malaria vignette, 68% (95% CI 61-75%) of CHWs indicated they would perform a rapid diagnostic test, and 75% (95% CI 66-83%) demonstrated this skill correctly.

CHWs are supposed to perform a MUAC assessment for all children regardless of presenting complaint. In the pneumonia and malaria vignettes, only 28% (95% CI 21-35%) and 30% (95% CI 24-38%) of CHWs indicated they would perform this assessment, respectively. Across all three vignettes, 43-65% of CHWs demonstrated the MUAC assessment correctly (on the QAO or another volunteer).

Supplementary Table 3: Advice to caregivers

**Task**	**% of CHWs performing task during pneumonia vignette (95% CI)**	**% of CHWs performing task during diarrhea vignette (95% CI)**	**% of CHWs performing task during malaria vignette (95% CI)**	**% of CHWs performing task in all 3 vignettes (95% CI)**	**% of vignettes during which task was performed (95% CI)**
Correctly counsels caregiver on how to give treatment	34 (25-41%)	41 (33-49%)	61 (53-68%)	25 (19-33%)	45 (40-50%)
Discusses follow-up with caregiver	35 (28-43%)	46 (39-54%)	56 (48-64%)	26 (19-33%)	46 (41-51%)
Provides preventive education	6 (3-12%)	6 (3-12%)	21 (15-28%)	2 (1-6%)	11 (8-14%)

Supplement to: Downey J, McKenna AH, Mendin SF, et al. Measuring knowledge of community health workers at the last mile in Liberia: feasibility and results of clinical vignette assessments. *Glob Health Sci Pract*. 2021;9(suppl 1). https://doi.org/10.9745/GHSP-D-20-00380

Overall, CHWs correctly counseled caregivers on home treatment in 45% (95% CI 40-50%) of vignettes. They also discussed follow-up with the caregiver in 46% (95% CI 41-51%) of vignettes. Preventive education was only provided in 11% (95% CI 8-14%) of vignettes. These tasks were all completed more often in the malaria vignette than in the other two vignettes, perhaps reflecting more familiarity with malaria cases than other case types. Approximately a quarter of CHWs correctly counseled caregivers and discussed follow up with caregivers in all three vignettes (25% and 26%, respectively), while only 2% (95% CI 1-6%) provided preventive education in all three vignettes.

Supplementary Table 4: Correct essential diagnosis, correct lifesaving treatment, and job aid use

	**Overall job aid use % (95% CI)**	**% correct diagnosis (95% CI)**			**% correct lifesaving treatment (95% CI)**		
		**Job aid used**	**Job aid not used**	***P* value**	**Job aid used**	**Job aid not used**	**Difference**
Pneumonia	43 (35-51%)	35 (23-46%)	20 (12-29%)	0.0206*	30 (19-41%)	18 (10-26%)	0.0362*
Diarrhea	43 (35-51%)	53 (41-65%)	20 (12-29%)	0.0000*	67 (55-78%)	37 (27-47%)	0.0001*
Malaria	43 (36-51%)	94 (88-100%)	73 (63-82%)	0.0003*	81 (71-90%)	53 (43-64%)	0.0002*
Total	43 (38-47%)						

** indicates statistical significance through a two-sample test of proportions, α=0.05.*

Overall, correct diagnosis and lifesaving treatment recommended by CHWs was higher among those who used job aids than among those who did not. For the pneumonia vignette, correct diagnosis and lifesaving treatment were 15 and 12 percentage points higher, respectively, among those who used job aids versus those who did not. For the diarrhea vignette, correct diagnosis and lifesaving treatment were 33 and 30 percentage points higher, respectively, among those who used job aids. For the malaria vignette, correct diagnosis and lifesaving treatment were 21 and 28 percentage points higher, respectively, among those who used job aids. These differences were statistically significant for all vignettes for both correct diagnosis and lifesaving treatment.
