# Supplementary material for: Measuring Knowledge of Community Health Workers at the Last Mile in Liberia: Feasibility and Results of Clinical Vignette Assessments
Source: Glob Health Sci Pract. 2021 Mar 15;9(Suppl 1):S111–21. doi: 10.9745/GHSP-D-20-00380 (PMC7971375; doi:10.9745/GHSP-D-20-00380)
Supplement: 20-00380-Downey-Supplement1.pdf [file 20-00380-Downey-Supplement1.pdf]

Supplement to: Downey J, McKenna AH, Mendin SF, et al. Measuring knowledge of community health workers at the last mile in Liberia: feasibility and results of clinical vignette assessments. Glob Health Sci Pract. 2021;9(suppl 1). https://doi.org/10.9745/GHSP-D-20-00380

LAST MILE HEALTH

Case Scenarios

Correct Treatment Rate Assessment

v1.7

These scenarios should be used by clinical quality assurance personnel in the field. Case scenarios should be read word for word to the CHA, and no information should be given to the CHA unless they ask for it (or unless otherwise stated). Forms should be submitted to RM&E within 2 weeks of the assessment taking place.

County:	Data collector name:
Health Facility:	CHA name:
CHA or CHSS being evaluated: ☐CHA ☐CHSS	CHA ID:
Community name:	CHSS name:
Community ID:	CHSS ID:
Date (dd-mm-yyyy):	

Read the following to the CHA:

> “The reason for these cases we will be reading to you is for us to see how you can do your everyday work.
>
> In each of the cases, the caregiver will come to you with a sick child. From there na, you start asking all your questions. You can use your job aid, paper forms, DCT, and all the tools you have to help you do your work.
>
> The same normal way you can do your work every day, that's how you should feel free in front of us. If you can always use the DCT first while asking the caregiver, do it. If that the paper forms first, do it.
>
> Ask any questions you want ask about the sick child. If you want to check the child, tell me what exactly you looking for and I will show you or tell you. We want you to know that you are in control and can decide whether to refer or not. All the drugs you need are in your box.
>
> Take your own time on each of the case, because when you are finished with one you will not go back to it.”

Before beginning the case scenarios, answer any questions the CHA may have. If the CHA makes mistakes during a case scenario, please note the mistakes and go over them with the CHA after ALL case scenarios have been performed.

Supplement to: Downey J, McKenna AH, Mendin SF, et al. Measuring knowledge of community health workers at the last mile in Liberia: feasibility and results of clinical vignette assessments. Glob Health Sci Pract. 2021;9(suppl 1). https://doi.org/10.9745/GHSP-D-20-00380

CASE SCENARIO 1

Read the following case scenario word-for-word to the CHA

"My daughter's stomach is running. She is eight months old. What will you do?"

Give the CHA time to look over the job aid and the sick child form (and to fill out a form, if they would like).

Check the box for each diagnostic activity the CHA completes WITHOUT PROMPTING, and give the CHA the information each activity provides. They may ask the questions in any order. If the CHA does not choose to complete certain activities, leave the checkbox blank.

Gathering information about the sick child

If the CHA asks an open-ended question, such as "Any other thing happened to the child?" you may give one additional piece of information about the child. If the CHA asks an open-ended question, please check the box below for "Any other questions asked" and write in the CHA's question.

- ☐ Has the child had fever? → "No"
- ☐ Has the child had cough? → "No"
- ☐ How long has the child had running stomach? → "She has had running stomach for 8 days."
- ☐ How many poopoo in one day? → "Four water poopoo in one day."
- ☐ Any blood in the stool? → "No, I don't think so."
- ☐ Can the child drink or breastfeed? → "She can be eating small small."
- ☐ Has the child been vomiting everything? → "No vomiting."
- ☐ Has the child has had any convulsions? → "No"
- ☐ Has the child already taken medication for this problem? → "No"
- ☐ Any other questions asked (*please write question asked here*):

Examining the sick child

- ☐ How does the child look? → "She looking tired."

Supplement to: Downey J, McKenna AH, Mendin SF, et al. Measuring knowledge of community health workers at the last mile in Liberia: feasibility and results of clinical vignette assessments. Glob Health Sci Pract. 2021;9(suppl 1). https://doi.org/10.9745/GHSP-D-20-00380

☐ Does the child feel hot? → "No, not too hot."

☐ Is the child very sleepy or unconscious? → "No, just weak small."

☐ Is there chest in-drawing? → *Show* **video 2** *of normal breathing*

☐ Yes ☐ No	Correctly identifies normal breathing

☐ Asks for respiratory rate → *Show* **video 2** *of normal breathing*
→ RR counted by CHA is ______.

☐ Yes ☐ No	Correctly counted respiratory rate **(Correct RR is 36 +/- 3)**

☐ Asks to check the child's MUAC → *ask for demonstration of skill if CHA has not already done so in prior case* → report that the MUAC is in the red zone.

☐ Yes ☐ No	Correctly demonstrates MUAC

☐ Is there swelling in the feet? → "No."

☐ Wants to perform RDT → *ask for demonstration of skill if CHA has not already done so in prior case* → report that result is negative

☐ Yes ☐ No	Correctly demonstrates RDT

☐ Any other exam questions of diagnostic activity (*please write question asked here*):

Diagnosis

Ask the CHA what the diagnosis is. Check all boxes that the CHA mentions WITHOUT PROMPTING. If the CHA mentions there is a danger sign present, ask which one and write it down on the line provided.

☐ Danger Sign (write in):		
☐ Diarrhea	☐ Simple Cough	☐ Pneumonia
☐ Simple Fever	☐ Malaria	☐ Malnutrition
☐ Child is healthy	☐ Other (write in):	

DO NOT CORRECT THE CHA IF HE/SHE MAKES THE WRONG DIAGNOSIS
CORRECT DIAGNOSIS: DIARRHEA; DANGER SIGN: RED MUAC (MALNUTRITION)

Ask the CHA how they would manage this child. Check all boxes that the CHA mentions WITHOUT PROMPTING. ***Please write the dosage, frequency/schedule, and duration the CHA prescribes in the spaces provided next to the drug name*** *(it is okay to prompt the CHA for dose, frequency and duration if they do not tell you up front). The correct items are in* ***bold.***

Treatment activities

☐ **Refers to the facility**

☐ **Initiated referral as soon as a danger sign was mentioned OR**

☐ Continued evaluation when danger signs was mentioned, but later made referral

☐ **Gives pre-referral treatment (*mark the treatment they select below*)**

☐ **ORS: __________ (dose) ________ (frequency) ________ (duration)**

☐ **Correct dosing for case** ☐ Dosing error made

☐ Zinc: __________ (dose) __________ (frequency) __________ (duration)

☐ Paracetamol: __________ (dose) __________ (frequency) __________ (duration)

☐ Amoxicillin: __________ (dose) __________ (frequency) __________ (duration)

☐ ACT: __________ (dose) __________ (frequency) __________ (duration)

☐ Artesunate suppository:________ (dose) _______ (frequency) _______ (duration)

CORRECT TREATMENT: ORS 1 SACHET, PREPARE 1 LITER AND REFER IMMEDIATELY TO FACILITY FOR MALNUTRITION

Did the CHA correctly counsel the caregiver in how to give the treatment?	☐ Yes ☐ No
Did the CHA discuss follow-up with the caregiver?	☐ Yes ☐ No
Did the CHA provide preventative education?	☐ Yes ☐ No

Did the CHA use their job aids, their DCT, or a paper form during the case scenario? Please check all that apply.

☐ Job Aid(s) ☐ DCT Form ☐ Paper Form

Any other notes:

Supplement to: Downey J, McKenna AH, Mendin SF, et al. Measuring knowledge of community health workers at the last mile in Liberia: feasibility and results of clinical vignette assessments. Glob Health Sci Pract. 2021;9(suppl 1). https://doi.org/10.9745/GHSP-D-20-00380

CASE SCENARIO 2

Read the following case scenario word-for-word to the CHA

"My grandson is coughing plenty. He is four years old. What will you do?"

Give the CHA time to look over the job aid and the sick child form (and to fill out a form, if they would like).

Check the box for each diagnostic activity the CHA completes WITHOUT PROMPTING, and give the CHA the information each activity provides. They may ask the questions in any order. If the CHA does not choose to complete certain activities, leave the checkbox blank.

Gathering information about the sick child

If the CHA asks an open-ended question, such as "Any other thing happened to the child?" you may give one additional piece of information about the child. If the CHA asks an open-ended question, please check the box below for "Any other questions asked" and write in the CHA's question.

- ☐ Has the child had fever? → "No"
- ☐ Has the child had running stomach? → "No"
- ☐ How long the child been coughing now? → "For two weeks now."
- ☐ He drinking or eating? → "Yes, he is drinking and eating."
- ☐ Has the child been vomiting everything? → "No, no vomiting."
- ☐ Is there any blood in the child's stool? → "No"
- ☐ Has the child has had any convulsions? → "No"
- ☐ Has the child already taken medication for this problem? → "No"
- ☐ Any other questions asked (*please write question asked here*):

Examining the sick child

- ☐ How does the child look? → "He is up and can look at you when you speak, but coughing fast, fast."
- ☐ Is the child very sleepy or unconscious? → "He is very weak, but still awake."

☐ Does the child feel hot? → "No, not too hot."

☐ Is there chest in-drawing? → *Show* **video 3** *of fast breathing, NO chest in-drawing, >1yo*

☐ Yes ☐ No	Correctly identifies normal breathing (NO in-drawing)

☐ Asks for respiratory rate → *Shows* **video 3** *of fast breathing, NO chest in-drawing, >1yo*

→ RR counted by CHA is ______.

☐ Yes ☐ No	Correctly counted respiratory rate (Correct RR is 92 +/- 3 (must be > 40))

☐ Asks to check the child's MUAC → *ask for demonstration of skill if CHA has not already done so in prior case* → report that the MUAC is in the green zone.

☐ Yes ☐ No	Correctly demonstrates MUAC

☐ Is there swelling in the feet? → "No."

☐ Wants to perform RDT → *ask for demonstration of skill if CHA has not already done so in prior case* → report that result is negative

☐ Yes ☐ No	Correctly demonstrates RDT

☐ Any other exam questions of diagnostic activity (*please write question asked here*):

Diagnosis

Ask the CHA what the diagnosis is. Check all boxes that the CHA mentions WITHOUT PROMPTING. If the CHA mentions there is a danger sign present, ask which one and write it down on the line provided.

☐ Danger Sign (write in):		
☐ Diarrhea	☐ Simple Cough	☐ Pneumonia
☐ Simple Fever	☐ Malaria	☐ Malnutrition
☐ Child is healthy	☐ Other (write in):	

DO NOT CORRECT THE CHA IF HE/SHE MAKES THE WRONG DIAGNOSIS

CORRECT DIAGNOSIS: PNEUMONIA; DANGER SIGN: COUGH FOR 14 DAYS

Supplement to: Downey J, McKenna AH, Mendin SF, et al. Measuring knowledge of community health workers at the last mile in Liberia: feasibility and results of clinical vignette assessments. Glob Health Sci Pract. 2021;9(suppl 1). https://doi.org/10.9745/GHSP-D-20-00380

Ask the CHA how they would manage this child. Check all boxes that the CHA mentions WITHOUT PROMPTING. ***Please write the dosage, frequency/schedule, and duration the CHA prescribes in the spaces provided next to the drug name*** *(it is okay to prompt the CHA for dose, frequency and duration if they do not tell you up front). The correct items are in* ***bold.***

Treatment activities

- ☐ **Refers to the facility**
  - ☐ **Initiated referral as soon as a danger sign was mentioned OR**
  - ☐ Continued evaluation when danger signs was mentioned, but later made referral
- ☐ **Gives pre-referral treatment (*mark the treatment they select below*)**
- ☐ ORS: __________ (dose) __________ (frequency) __________ (duration)
- ☐ Zinc: __________ (dose) __________ (frequency) __________ (duration)
- ☐ Paracetamol: __________ (dose) __________ (frequency) __________ (duration)
- ☐ **Amoxicillin:_________(dose)_________ (frequency)_________ (duration)**
  - ☐ **Correct dosing for case** ☐ Dosing error made
- ☐ ACT: __________ (dose) __________ (frequency) __________ (duration)
- ☐ Artesunate suppository: _______ (dose) ________ (frequency) _______ (duration)

CORRECT TREATMENT: REFER TO HEALTH FACILITY AND GIVE AMOXICILLIN 250MG, 2 TABLETS, ONCE PREREFERAL

Did the CHA correctly counsel the caregiver in how to give the treatment?	☐ Yes ☐ No
Did the CHA discuss follow-up with the caregiver?	☐ Yes ☐ No
Did the CHA provide preventative education?	☐ Yes ☐ No

Did the CHA use their job aids, their DCT, or a paper form during the case scenario? Please check all that apply.

☐ Job Aid(s)	☐ DCT Form	☐ Paper Form

Any other notes:

Supplement to: Downey J, McKenna AH, Mendin SF, et al. Measuring knowledge of community health workers at the last mile in Liberia: feasibility and results of clinical vignette assessments. Glob Health Sci Pract. 2021;9(suppl 1). https://doi.org/10.9745/GHSP-D-20-00380

CASE SCENARIO 3

Read the following case scenario word-for-word to the CHA

"My daughter is weak. She is two years old. What will you do?"

Give the CHA time to look over the job aid and the sick child form (and to fill out a form, if they would like).

Check the box for each diagnostic activity the CHA completes WITHOUT PROMPTING, and give the CHA the information each activity provides. They may ask the questions in any order. If the CHA does not choose to complete certain activities, leave the checkbox blank.

Gathering information about the sick child

If the CHA asks an open-ended question, such as "Any other thing happened to the child?" you may give one additional piece of information about the child. If the CHA asks an open-ended question, please check the box below for "Any other questions asked" and write in the CHA's question.

- ☐ Is the child's skin hot? → "Yes"
- ☐ Has the child had running stomach? → "No"
- ☐ Has the child had cough? → "No"
- ☐ Ask how long the child has had a fever → "Her skin been hot for 4 days now."
- ☐ Can the child drink or breastfeed? → "Yes, she is eating ok."
- ☐ Has the child been vomiting everything? → "No vomiting."
- ☐ Is there any blood in the child's stool? → "No"
- ☐ Has the child had any convulsions? → "No"
- ☐ Has the child already taken medication for this problem? → "No"
- ☐ Any other questions asked (*please write question asked here*):

Examining the sick child

- ☐ Does the child feel hot? → "Yes, the skin is very hot."
- ☐ How does the child look? → "She looking weak, but fine."

Supplement to: Downey J, McKenna AH, Mendin SF, et al. Measuring knowledge of community health workers at the last mile in Liberia: feasibility and results of clinical vignette assessments. Glob Health Sci Pract. 2021;9(suppl 1). https://doi.org/10.9745/GHSP-D-20-00380

☐ Is the child very sleepy or unconscious? → "No, just very tired."

☐ Is there chest in-drawing? → *Show* ***video 2*** *of normal breathing*

☐ Yes ☐ No	Correctly identifies normal breathing

☐ Asks for respiratory rate → *Show* ***video 2*** *of normal breathing*
→ RR counted by CHA is ______.

☐ Yes ☐ No	Correctly counted respiratory rate (Correct RR is 36 +/- 3)

☐ Asks to check the child's MUAC → *ask for demonstration of skill if CHA has not already done so in prior case* → report that the MUAC is in the green zone

☐ Yes ☐ No	Correctly demonstrates MUAC

☐ Is there swelling in the feet? → "No."

☐ Wants to perform RDT → *ask for demonstration of skill if CHA has not already done so in prior case* → report that result is POSITIVE

☐ Yes ☐ No	Correctly demonstrates RDT

☐ Any other exam questions of diagnostic activity (*please write question asked here*):

Diagnosis

Ask the CHA what the diagnosis is. Check all boxes that the CHA mentions WITHOUT PROMPTING. If the CHA mentions there is a danger sign present, ask which one and write it down on the line provided.

☐ Danger Sign (write in):		
☐ Diarrhea	☐ Simple Cough	☐ Pneumonia
☐ Simple Fever	☐ Malaria	☐ Malnutrition
☐ Child is healthy	☐ Other (write in):	

DO NOT CORRECT THE CHA IF HE/SHE MAKES THE WRONG DIAGNOSIS

CORRECT DIAGNOSIS: MALARIA; DANGER SIGN: NONE

Ask the CHA how they would manage this child. Check all boxes that the CHA mentions WITHOUT PROMPTING. **Please write the dosage, frequency/schedule, and duration the CHA prescribes in the spaces provided next to the drug name** *(it is okay to prompt the CHA for dose, frequency and duration if they do not tell you up front). The correct items are in* **bold.**

Treatment activities

- ☐ Refers to the facility
  - ☐ Initiated referral as soon as a danger sign was mentioned OR
  - ☐ Continued evaluation when danger signs was mentioned, but later made referral
- ☐ Gives pre-referral treatment (*mark the treatment they select below*)
- ☐ ORS: __________ (dose) __________ (frequency) __________ (duration)
- ☐ Zinc: __________ (dose) __________ (frequency) __________ (duration)
- ☐ **Paracetamol:_________ (dose) __________ (frequency) _________(duration)**
  - ☐ **Correct dosing for case** ☐ Dosing error made
- ☐ Amoxicillin: __________ (dose) __________ (frequency) __________ (duration)
- ☐ **ACT: __________ (dose) __________ (frequency) __________ (duration)**
  - ☐ **Correct dosing for case** ☐ Dosing error made
- ☐ Artesunate suppository: _______ (dose) ________ (frequency) _______ (duration)

CORRECT TREATMENT: ACT 50, 1 TABLET 1x/DAY x 3 DAYS and PARACETAMOL 100 MG 2 TABLETS 3x/DAY x 3 DAYS

Did the CHA correctly counsel the caregiver in how to give the treatment?	☐ Yes ☐ No
Did the CHA discuss follow-up with the caregiver?	☐ Yes ☐ No
Did the CHA provide preventative education?	☐ Yes ☐ No

Did the CHA use their job aids, their DCT, or a paper form during the case scenario? Please check all that apply.

☐ Job Aid(s)	☐ DCT Form	☐ Paper Form

Any other notes:
