# Supplementary material for: Measuring Knowledge of Community Health Workers at the Last Mile in Liberia: Feasibility and Results of Clinical Vignette Assessments
Source: Glob Health Sci Pract. 2021 Mar 15;9(Suppl 1):S111–21. doi: 10.9745/GHSP-D-20-00380 (PMC7971375; doi:10.9745/GHSP-D-20-00380)
Supplement: 20-00380-Downey-Supplement3.pdf [file 20-00380-Downey-Supplement3.pdf]

Supplement to: Downey J, McKenna AH, Mendin SF, et al. Measuring knowledge of community health workers at the last mile in Liberia: feasibility and results of clinical vignette assessments. *Glob Health Sci Pract*. 2021;9(suppl 1). https://doi.org/10.9745/GHSP-D-20-00380

Title, Author, Journal, & Year	Country / Region and Study Design	Conditions Assessed	Assessment Method(s)	Correct Diagnosis / Treatment Proportions	CHW Selection & Training	Definition of Correct Diagnosis / Treatment
**Community health worker competency in managing ARIs of childhood in Bolivia**<br><br>Zeitz et al.<br><br>Bulletin of the Pan American Health Organization (1993)	Rural Bolivia<br><br>Before and after cross-sectional (before and after delivery of a training course)	Acute respiratory infection	Role play using dolls to represent children and actors playing mothers; oral clinical vignettes / case scenarios; video case scenarios<br><br>Overall knowledge score derived from KAP survey variables	**Before course:**<br>Correct classification of ARI: 60%<br>Correct treatment knowledge: 34%<br><br>**After course:**<br>Correct classification of ARI: 83%<br>Correct treatment knowledge: 76%	CHWs were previously trained with WHO guidelines. The groups of CHWs were chosen because the areas they served had high incidence of ARI-associated childhood mortality. CHWs received between 1-2 weeks of training on ARI case management guidelines.	Competently use the WHO guidelines, which included the following: (1) history taking, (2) physical evaluation, (3) disease classification, (4) assignment of treatment, (5) use of medication, (6) education of mothers, (7) patient follow-up, (8) record keeping.
**ARIs in Nigerian children: Prospective cohort study of incidence and case management**<br><br>Fagbule et al.<br><br>Journal of Tropical Pediatrics (1994)	Kwara State, Nigeria<br><br>Community-based prospective surveillance and case management study	Acute respiratory infection	Register review: thrice-weekly surveillance of a cohort of children living in the study area with active CHWs trained in ARI diagnosis and treatment, to check for ARI morbidity and mortality	Mild: 81% correct treatment<br>Moderate: 71% correct treatment (11% referred unnecessarily, 18% did not receive antibiotics)<br>Severe: 100% referred (correct treatment)	CHWs were proficient in the local language and had 3 years of basic health training at the School of Health Technology in Nigeria. All CHWs completed a 2-week intensive training on WHO ARI guidelines.	ARI classifications were as follows: Mild ARI = Cough + RR < 50/min<br>Moderate ARI = Cough + RR> 50/min<br>Severe ARI = Cough + RR > 50/min + Chest in-drawing<br><br>Oral adult cotrimoxazole tablet (dose: from 2 months to 12 months—1/2 tablet twice daily for 5 days; from 12 months to 5 years—1 tablet twice daily for 5 days) was used as the first line antibiotic treatment.
**Improving skills and utilization of CHVs in Nepal**<br><br>Curtale et al.<br><br>Social Science & Medicine (1995)	Rural Nepal<br><br>Evaluation of a nutrition education intervention	Malnutrition, diarrhea, acute respiratory infection	Caretaker interview (household survey, asked mothers about child illnesses in past 2 weeks and any treatment by CHVs)	**Intervention:**<br>Treatment with ORS: 78%<br>Treatment of ARI: 37%<br><br>**Control:**<br>Treatment with ORS: 43%<br>Treatment of ARI: <1%	CHWs were volunteer, local women who were trained for 24 days.	Ability to identify night blindness, citing lack of dark green leafy vegetables as cause of night blindness, recognition of fast breathing as major sign of ARI, and recognition of moderate to severe cases of ARI and treatment with co-trimoxazole.
**Community Health Worker Performance in the Management of Multiple Childhood Illnesses: Siaya District, Kenya, 1997-2001**<br><br>Kelly et al.<br><br>American Journal of Public Health (2001)	Siaya District, Kenya<br><br>3 cross-sectional, hospital-based evaluations	Pneumonia, malaria, dehydration	CHWs invited to Siaya District Hospital and asked to manage up to 4 ill children<br><br>Methods for data collection included observation of consultations using a checklist; CHW documentation of the assessment findings, classifications, treatments for each child on a standard form; exit interviews with caregivers; repeat examination of children by a study clinician (direct observation and re-examination, register review, and exit interviews)	**First study / second study / third study:**<br><br>CHW recognizes 1+ danger signs: 62.8% / 67.4% / 54.7%<br><br>Severe classifications correctly identified: 49.4% / 44.7% / 51.1%<br><br>Pneumonia correctly identified: 33.3% / 54.5% / 31.5%<br><br>Malaria correctly identified: 41.3% / 67.8% / 63.9%<br><br>Pneumonia, adequate treatment: 58.3% / 65.1% / 50.0%<br><br>Pneumonia, recommended treatment: 58.3% / 57.6% / 39.4%<br><br>Malaria, adequate treatment: 41.0% / 92.7% / 90.5%	CHWs were volunteers that were literate and selected by their communities. CHWs were trained using a version of the IMCI guidelines. The CHWs received 3 weeks of initial training and then refresher trainings throughout. Refresher trainings lasted for a week.	Definitions for correct classification, treatment, and referral were based on the CARE algorithm.<br>Danger signs were inability to drink or breastfeed, convulsions, “vomiting everything,” and lethargy or unconsciousness. “Gold standard” classifications and treatments were generated with a computer program that applied the CARE guidelines to the assessment findings from the study clinician’s repeat examination.<br><br>Treatment was defined as “recommended” (all indicated medications were selected and children with a severe classification were referred) or “adequate” (antibiotic, antimalarial, or oral rehydration solution, as indicated, was selected, plus referral for severe classifications).<br><br>Recommended and adequate treatments, defined as correct drug selection, did not consider drug dosing. When both correct

Title, Author, Journal, & Year	Country / Region and Study Design	Conditions Assessed	Assessment Method(s)	Correct Diagnosis / Treatment Proportions	CHW Selection & Training	Definition of Correct Diagnosis / Treatment
				Malaria, recommended treatment: 40.0% / 69.3% / 60.5%<br><br>Correct referral: 70.0% / 42.1% / 52.7%<br><br>Adequate treatment for severe illness: 57.8% / 35.5% / 38.9%<br><br>Recommended treatment for severe illness: 54.4% / 28.9% / 30.5%		drug selection and dosing were required for treatment to be considered correct, treatment scores were lower.
**Diagnosis of pneumonia by community health volunteers: experience of BRAC, Bangladesh**<br><br>Hadi<br><br>Tropical Doctor (2001)	10 sub-districts in northern and central regions of Bangladesh<br><br>Cross-sectional survey	Pneumonia	Direct observation and re-examination	Sensitivity of diagnosis: 67.7%<br>Specificity of diagnosis: 95.2%	CHWs were part time and had formal schooling. They received pre and in-service training in diagnosing and treating children with ARI. Some of the CHWs has been working for more than 5 years.	Diagnosis was based on the WHO guidelines. These included measurement of respiratory rate, assessment of chest in-drawing, noisy breathing, body temperature, shrunken eyes and a history of convulsion. A child was considered to have a raised respiratory rate if: (i) more than 50 breaths/min were counted in children aged 2-11 months; and (ii) more than 40 breaths/min were counted in children between 12 and 60 months. The severity of pneumonia was graded as very severe, severe, mild and none.<br><br>Specificity, sensitivity and PPV of CHWs' diagnosis of pneumonia compared with that of research physicians were calculated through 2 x 2 contingency tables.
**Management of acute respiratory infections by community health volunteers: Experience of Bangladesh Rural Advancement Committee (BRAC)**<br><br>Hadi<br><br>Bulletin of the World Health Organization (2003)	10 sub-districts in Bangladesh<br><br>Cross-sectional study	Acute respiratory infection	Direct observation and re-examination	Sensitivity of diagnosis: 67.7%<br>Specificity of diagnosis: 95.2%<br><br>Overall proportion of agreement for diagnosis: 89%<br>Overall proportion of agreement for treatment: 87.2%	CHWs were community health volunteers at the grass roots level. They were selected in their local communities and most only had 5 years of schooling. CHWs underwent extensive 3-day training with a group of physicians and para-professionals with experience in managing and treating ARIs. There was routine refresher training.<br><br>Sometimes it was not possible to organize a 3-day formal basic training for new volunteers so every month a special training day was organized where most topics of the basic training was taught.	The diagnosis was categorized into very severe, severe, and mild cases. A case was considered very severe if the child was not able to drink, had convulsions, or looked abnormally sleepy; if there was stridor in a calm child; or if the child was severely malnourished. If no sign of a very severe case was identified, but the child had chest in-drawing, the child concerned was diagnosed as having a severe ARI. The case was considered mild if the child had no signs of very severe or severe infection but was considered to have a respiratory rate >40 breaths per minute.
**Can community health workers and caretakers recognize pneumonia in children?**	Western Uganda<br><br>Cross-sectional mixed methods	Acute respiratory infection	Direct observation and re-examination	Correct classification: 79%	CHWs in this study were drug distributers (DDs) who underwent a 2-day training by a nurse and a clinical officer using IMCI methodology.	CHWs were evaluated for their capacity to assess breathing rate by blind assessment of three children with clinically verified pneumonia with fast breathing and three children with normal respiratory rate (RR).

Supplement to: Downey J, McKenna AH, Mendin SF, et al. Measuring knowledge of community health workers at the last mile in Liberia: feasibility and results of clinical vignette assessments. *Glob Health Sci Pract*. 2021;9(suppl 1). https://doi.org/10.9745/GHSP-D-20-00380

Title, Author, Journal, & Year	Country / Region and Study Design	Conditions Assessed	Assessment Method(s)	Correct Diagnosis / Treatment Proportions	CHW Selection & Training	Definition of Correct Diagnosis / Treatment
**Experiences from western Uganda**<br><br>Kallander et al.<br><br>Transactions of the Royal Society of Tropical Medicine and Hygiene (2005)						Fast breathing was defined according to the IMCI criteria: ≥50 breaths/min for 2—11 months and ≥40 breaths/min for 12—59 months. CHWs were asked: (1) to judge whether the child's RR appeared fast or normal by impression; (2) to count the RR for 1 min; and (3) to classify the child as fast or normal according to age-specific cut-offs.
**Effect of multiple interventions on CHWs' adherence to clinical guidelines in Siaya district, Kenya**<br><br>Rowe et al.<br><br>Transactions of the Royal Society of Tropical Medicine and Hygiene (2007)	Siaya District, Kenya<br><br>Cross-sectional survey	Pneumonia, malaria, diarrhea, measles	Direct observation and re-examination	Overall treatment with no error / minor error / major error: 38.8% / 19.0% / 42.2%<br><br>Overall guideline adherence per child: 80% of all guideline-recommended procedures performed correctly on average<br><br>Overall prescription of potentially life-saving treatments including referral: 58% of ill children	CHWs were required to read at "standard 7" school level (equivalent to being an elementary school graduate in USA), volunteer, and reside in the community. Some villages had health committees that selected the CHWs where they solicited opinions from women in the community. Initial and refresher trainings were provided.	Overall guideline adherence score calculated involving 29 procedures (18 assessment tasks, 4 illness classifications, 7 treatments including referral, all equally weighted, not all required for each child).<br><br>Analyzed treatment with no error vs. treatment with a minor or major error, and treatment with major error vs treatment with no error or minor error.
**Longitudinal analysis of community health workers' adherence to treatment guidelines, Siaya, Kenya, 1997-2002**<br><br>Rowe et al.<br><br>Tropical Medicine and International Health (2007)	Siaya District, Kenya<br><br>Longitudinal study	Pneumonia, malaria, dehydration	Same cohort of CHWs as analyzed in Kelly et al. (2001), but this study used only register review	55.8% of consultations were prescribed all recommended treatments<br>Mean CHW adherence to guidelines score: 79.4%	CHWs served the communities that they came from. The study cohort looked at the same CHWs after different amounts of training.	The outcome was an adherence score, defined as the percentage of guideline-recommended treatments pre-scribed for each patient. This score was a continuous variable ranging from 0% to 100% and encompassed seven treatments: five oral drugs, one antibiotic eye ointment and referral to a health facility if indicated. The adherence score measured the CHW's ability to process clinical information to choose recommended treatments. The adherence score for each ill child was calculated according to the guidelines that existed at the time of the consultation.
**Assessing the quality of service of village malaria workers to strengthen community-based malaria control in Cambodia**<br><br>Yasuoka et al.<br><br>Malaria Journal (2010)	7 remote provinces of Cambodia<br><br>Cross-sectional study	Malaria	Interviews with village malaria workers (self report)	Service quality: mean 3.174 (out of 5)<br>Actions for malaria prevention and vector control: mean 12.833 (out of 23)	VMWs participated in a 3 day training program that covers malaria epidemiology, prevention, diagnosis using RDTs, treatment with artesunate, and mefloquine, referral to hospitals, and recording of fever cases and positive RDT results.<br><br>Trained VMWs are supposed to perform RDTs on any villager suspected of having malaria and, for test-positive cases, provide blister-packaged A+M according to the national guidelines. They are also encouraged to conduct active case detection, followup patients, and spread	Two additive indices to quantify service quality and actions, developed based on respondent answers to questions, including the items listed below.<br><br>**Service quality:**<br>Active detection<br>Diagnosis and treatment<br>Prescription of anti-malarials<br>Follow-up<br>Dissemination of preventive measures<br><br>**Actions:**<br>Malaria preventive measures<br>Vector control measures

Title, Author, Journal, & Year	Country / Region and Study Design	Conditions Assessed	Assessment Method(s)	Correct Diagnosis / Treatment Proportions	CHW Selection & Training	Definition of Correct Diagnosis / Treatment
					information on preventive measures to their villagers.	
**Can lay community health workers be trained to use diagnostics to distinguish and treat malaria and pneumonia in children? Lessons from rural Uganda**<br><br>Mukanga et al.<br><br>Tropical Medicine and International Health (2011)	Iganga district, Uganda<br><br>Cross-sectional study	Malaria and pneumonia	Direct observation and re-examination	96.7% adequate performance in taking history<br><br>96.0% adequate performance in using timer for respiratory rate<br><br>96.3% adequate use of RDTs<br><br>86.8% adequate classification<br><br>95.5% correct antimalarial prescription<br><br>40% correct antibiotic prescription<br><br>90.6% correct antibiotic / antimalarial prescription	None given	**Four indicators were used to assess ability to use a respiratory timer including:**<br>Counting rate before taking off blood<br>Ensuring child is settled before beginning to count<br>Following instructions (looks at child's lower part of the chest, start the timer by pressing center circle, start counting at the beep and stop counting after two beeps indicating a minute) on how to take the count and recording rate.<br><br>**Fourteen indicators were used to assess RDT use:**<br>Ensuring all inputs required are available before start<br>Correctly wearing gloves<br>Selecting correct finger to puncture<br>Cleaning finger with alcohol swab<br>Allowing finger to dry<br>Puncturing finger correctly<br>Drawing blood at this point using a pipette<br>Wiping finger with cotton after Collecting blood<br>Labelling child's ID number on cassette<br>Putting five drops of buffer into appropriate hole<br>recording time after adding buffer<br>waiting 15 min after adding buffer to read results and recording test results.<br><br>Classification ⁄ diagnosis made by CHW, and treatment prescribed were compared with that by the pediatrician
**Community case management of malaria using ACT and RDT in two districts in Zambia: Achieving high adherence to test results using community health workers**<br><br>Chanda et al.<br><br>Malaria Journal (2011)	Chongwe and Kalomo districts, Zambia<br><br>Prospective study	Malaria	Register review, focus group discussions, in-depth interviews	Uncomplicated malaria: 100%<br><br>0.6% overtreatment (gave antimalarials with negative RDT)<br><br>Referral for severe malaria: 100%	CHWs received standard training on malaria management based on national guidelines.	Using the diagnostic result to inform the decision whether to prescribe an anti-malarial or not for uncomplicated malaria, referral mechanisms for severe malaria cases and non-malaria febrile cases.

Supplement to: Downey J, McKenna AH, Mendin SF, et al. Measuring knowledge of community health workers at the last mile in Liberia: feasibility and results of clinical vignette assessments. *Glob Health Sci Pract*. 2021;9(suppl 1). https://doi.org/10.9745/GHSP-D-20-00380

Title, Author, Journal, & Year	Country / Region and Study Design	Conditions Assessed	Assessment Method(s)	Correct Diagnosis / Treatment Proportions	CHW Selection & Training	Definition of Correct Diagnosis / Treatment
**Comparison of Methods for Assessing Quality of Care for Community Case Management of Sick Children: An Application with CHWs in Malawi**<br><br>Cardemil et al.<br><br>American Journal of Tropical Medicine & Hygiene (2012)	Hard-to-reach areas in 6 districts in Malawi<br><br>Cross-sectional survey	Cough and fast breathing, fever, diarrhea	Direct observation and re-examination, direct observation only, register review, and case scenarios	**Uncomplicated illness % agreement (direct observation and re-examination):**<br>Fast breathing, classification / treatment: 77% / 73%<br>Fever, classification / treatment: 95% / 89%<br>Diarrhea, classification / treatment: 97% / 96%<br><br>**Severe illness % agreement (direct observation and re-examination):**<br>Fast breathing, classification / treatment: 81% / 45%<br>Fever, classification / treatment: 88% / 44%<br>Diarrhea, classification / treatment: 79% / 43%	HSAs are paid CHWs who are required to have a 10th grade education. HSAs underwent community case management training course that lasted for 6 days. HSAs were taught to recognize signs and symptoms and referred to the nearest health facility.	Analyses focused on three classifications: cough with fast breathing [suspected pneumonia and referred to as fast breathing throughout this report], fever [suspected malaria], and diarrhea. Cases were divided into uncomplicated illness and severe illness, the latter defined as any illness with danger signs<br><br>Uncomplicated illness: includes cough and fast breathing, diarrhea, or fever without any danger signs.<br><br>Severe illness: includes fast breathing, diarrhea, or fever with one more danger signs requiring referral according to CCM guidelines. Danger signs include: chest indrawing, cough for more than 21 days, fever for more than 7 days, diarrhea for more than 14 days, blood in stool, inability to drink or feed, vomits everything, convulsions, palmar pallor, red or mid-upper arm circumference tape, or bipedal edema.
**Integrated community case management of fever in children under five using rapid diagnostic tests and respiratory rate counting: A multi-country cluster randomized trial**<br><br>Mukanga et al.<br><br>American Journal of Tropical Medicine & Hygiene (2012)	Burkina Faso, Ghana, Uganda<br><br>Open cluster-randomized two-arm trial to assess effect of use of diagnostic and treatment package for iCCM comprising RDTs/ACTs for malaria, RRTs / antibiotics for pneumonia on recovery from fever, and rational use of meds	Malaria, pneumonia	Register review over the course of the study - QA through weekly visits by field supervisors including completeness of registers, drug administration, and assessment / follow-up of enrolled children; monthly review meetings for all CHWs with support / retraining if necessary where CHWs demonstrated their competency with performing and reading RDTs	Burkina Faso:<br>RDT+, high RR: 81.5%<br>RDT+, normal RR: 64.7%<br>RDT-, high RR, cough: 94.3%<br>RDT-, normal RR: 33.3%<br><br>Ghana:<br>RDT+, high RR: 70.8%<br>RDT+, normal RR: 60.2%<br>RDT-, high RR: 91.7%<br>RDT-, normal RR: 31.3%<br><br>Uganda:<br>RDT+, high RR: 97.8%<br>RDT+, normal RR: 99.0%<br>RDT-, high RR: 97.1%<br>RDT-, normal RR: 85.4%	CHWs were selected by their respective community. All CHWs needed to be able to read and write clearly. Burkina Faso: new CHWS were selected. Uganda: existing CHWs were used that were previously trained in providing anti-malarial drugs in the community. Ghana: existing community health nurses were used.<br><br>CHWs were taught how to take history, recognize clinical features of uncomplicated malaria, and signs of severe illness requiring referral; preparation of thick blood films for malaria microscopy; use of classification and treatment algorithms for malaria and pneumonia (intervention arm only); use of simple dosing guidelines based on age for ACTs and PCT; managing drug supplies; obtaining informed consent; and completing CRFs including documentation of reported signs and symptoms, physical examination results, and medications administered to the child.	Accuracy of CHWs performing RDTs, including how to correctly do the finger prick and read the result; microscopy was used to establish accuracy of results in the community.<br><br>No other definitions given.

Title, Author, Journal, & Year	Country / Region and Study Design	Conditions Assessed	Assessment Method(s)	Correct Diagnosis / Treatment Proportions	CHW Selection & Training	Definition of Correct Diagnosis / Treatment
**Performance of CHWs under iCCM of childhood illnesses in eastern Uganda**<br>Kalyango et al.<br>Malaria Journal (2012)	Eastern Uganda<br>Cross-sectional mixed methods	Malaria, pneumonia	Knowledge tests, case scenarios, register review, direct observation and re-examination, caregiver interviews	Register review measurement competency: 49% within 5 breaths of the gold standard clinician; 89% correctly categorized the breathing rates they obtained into pneumonia / not pneumonia<br>Classification: 75% correct for pneumonia<br>(classification and treatment numbers otherwise unavailable - only figures comparing study arms)	CHWs were selected from the communities where they live and work and underwent short-term training. All CHWs received training on malaria for three days. The CHWs in the dual-management arm received 3 more days of training in ARI. All CHWs received training on referrals. 13% had at most a primary education; 38% had at most a secondary education; 5% had at most a tertiary education.	CHWs diagnose malaria and pneumonia according to IMCI classification of illness. Children are classified as having “malaria” if they have fever or history of fever within the previous 24 hours; and as having “pneumonia” if they have cough and difficult breathing or fast breathing (≥50 breaths per minute in children aged four to 12 months and ≥40 breaths per minute in children 12 to 59 months). A diagnosis of severe disease is made if the child has any of the four general danger signs: convulsions, repeated vomiting, lethargy / unconsciousness or failure to feed, or other danger signs: chest in-drawings, noisy breathing, severe dehydration and pallor. CHWs should follow up children that have been treated and refer those that did not get well to the nearest health unit.
**Quality and safety of integrated community case management of malaria using rapid diagnostic tests and pneumonia by community health workers**<br>Hamer et al.<br>Pathogens and Global Health (2012)	Rural Zambia<br>Cluster-randomized controlled trial of two models for community-based management of malaria and pneumonia	Malaria, pneumonia	Register review, patient follow up<br>(CHWs also visited health centers monthly for performance assessment, which included observation of consultations by health center manager, who kept monthly record of performance and any CHW not performing satisfactorily was provided with remedial instruction)	RDTs done for 95.9% of children enrolled<br>**Correct classification - clinical feature (expected or correct classification in parentheses - since this was register review there was no evaluation of the correctness of clinical features):**<br>RDT+ (malaria or both malaria and pneumonia): 99.6%<br>RDT- (pneumonia or fever): 99.7%<br>Fast breathing (pneumonia or both malaria and pneumonia): 98.3%<br>No fast breathing (malaria or fever): 100%<br>RDT+ and fast breathing (malaria and pneumonia): 97.1%<br>RDT+ and no fast breathing (malaria): 100%<br>RDT- and fast breathing (pneumonia): 93.3%<br>RDT- and no fast breathing (fever): 100%<br>Correct prescription - RDT results / fast breathing (correct treatment in parentheses):<br>RDT+ (AL): 98.5%<br>RDT- (no AL): 99.6%<br>RDT+ and fast breathing (AL and amox): 93.2%<br>RDT- and fast breathing (amox): 98.3%<br>Fast breathing (amox): 98.1%	Most (72.2%) of CHWs had completed secondary school education. CHWs participated in a 5-day training session where they were taught to classify and treat children with malaria and/or pneumonia according to a simple algorithm.	The details and steps of the algorithm was the following: the first step was for the CHW to identify children with danger signs and then to refer them to the nearest rural health center. Danger signs were convulsions, coma/drowsiness, scanty urine output, pallor suggestive of severe anemia, deep jaundice, dark colored urine, persistent vomiting, chest wall in-drawing, noisy breathing, severe difficulty breathing, abnormally sleepy, not feeding, and inability to drink.<br>The CHW asked the child’s caregiver about specific complaints, when the signs of infection appeared, and medication history. They did a basic examination including counting respiratory rate (RR) and measuring temperature and weight. A RDT was performed, following which the CHW classified the child as having malaria, malaria and pneumonia, pneumonia, or RDT-negative fever. RR of >50 breaths per minute in children ,12 months and >40 breaths per minute in children >12 months was considered elevated and suggestive of pneumonia. Children were classified with malaria, if the RDT result was positive and the RR was normal; with malaria and pneumonia, if the RDT result was positive and the RR was increased; with pneumonia, if the RDT result was negative and the RR was high;

Title, Author, Journal, & Year	Country / Region and Study Design	Conditions Assessed	Assessment Method(s)	Correct Diagnosis / Treatment Proportions	CHW Selection & Training	Definition of Correct Diagnosis / Treatment
				No fast breathing (no amox): 98.6%<br>RDT- and no fast breathing (no AL, no amox): 98.3%		or with RDT-negative fever, if the RDT result was negative and RR was normal
**Assessment of the adherence of community health workers to dosing and referral guidelines for the management of fever in children under 5 years: a study in Dangme West District, Ghana**<br><br>Chinbuah et al.<br><br>International Health (2013)	Dangme West District, Ghana<br><br>Study within a cluster-randomized, stepped-wedge, controlled, open trial measuring impact on child mortality of use of antimalarial (AAQ) vs antimalarial plus antibiotic (AAQ + AMX) for treatment of fever	Fever, malaria	Register review	**AAQ arm:**<br>Artesunate / amodiaquine (2-11 months; 12-59 months): 96.7%; 99.1% appropriate drugs for age<br><br>**AAQ + AMX arm:**<br>Artesunate / amodiaquine (2-11 months; 12-59 months): 98.9%; 98.8% appropriate drugs for age<br>Amoxicillin (2-11 months; 12-35 months; 36-59 months): 96.7%; 99.1%; 95.2% appropriate drugs for age<br><br>Artesunate / amodiaquine AND amoxicillin together (2-11 months; 12-59 months; 36-59 months): 98.7%; 91.6%; 94.9% appropriate drugs for age	CHWs were "lay persons" who have no training in formal health care education. They were selected from the communities they lived in, which were often >5km away from a health facility. CHWs were volunteers and not paid. CHWs were given a 3 day training on assessing, treating, and referring a child appropriately. CHWs were trained to treat children with fever with either antimalarial drug or with the antibiotic drug amoxicillin with home management of malaria strategy. Training field supervisors provided additional support to the CHWS.	Existing national and IMCI guidelines were used to develop CHW reference material and data collection forms. CHWs had to identify the warning signs based on the reference material and then refer them out.
**Quality of sick child care delivered by Health Surveillance Assistants in Malawi**<br><br>Gilroy et al.<br><br>Health Policy & Planning (2013)	6 districts in Malawi<br><br>Cross-sectional survey	Malaria / fever, diarrhea, pneumonia	Direct observation and re-examination, caregiver interviews, register review	Overall correct classification: 44%<br>Overall correct classification for uncomplicated illnesses: 68%<br><br>Uncomplicated fever / malaria (did not assess using RDT - HSAs were supposed to just treat with antimalarial if child had uncomplicated fever): 79%<br>Uncomplicated cough / fast breathing: 52%<br>Uncomplicated diarrhea: 69%<br><br>Correct referral: 55%<br>Correct pre-referral treatment AND correct referral: 52%	HSAs are employed under the Environmental Health section of the Ministry of Health. Recruitment criteria include literacy and completion of at least 2 years of secondary school. Each HSA undergoes a 10 week training program.	Correct case management was defined based on existing consensus indicators for the quality of sick children care developed for IMCI and adapted to refect Malawi CCM clinical guidelines.<br><br>**Definition of correct assessment for CCM-treatable illness**<br>Assessment for uncomplicated fever: HSA asked caregiver about the presence or history of fever or caregiver offered this complaint.<br>Assessment for cough with fast breathing: HSA asked caregiver about the presence of cough or caregiver offered this complaint; HSA asked about number of days of cough; HSA counted the child's respiratory rate using a timing device (e.g. timer or watch).<br>Assessment for diarrhoea: HSA asked caregiver about the presence of diarrhoea or caregiver offered this complaint; the number of days with diarrhoea and the presence of blood in stool.<br><br>**Definition of CCM-treatable illnesses**<br>Uncomplicated fever: Child with fever lasting less than 7 days without any danger signs.<br>Uncomplicated cough with fast breathing: Child with cough for less than 21 days with fast breathing (respiratory rate of 50

Title, Author, Journal, & Year	Country / Region and Study Design	Conditions Assessed	Assessment Method(s)	Correct Diagnosis / Treatment Proportions	CHW Selection & Training	Definition of Correct Diagnosis / Treatment
						breaths per minute or more for ages 2–12 months and 40 breaths per minute for ages 12 months to 5 years) without any danger signs.<br>Uncomplicated diarrhoea: Child with diarrhoea less than 14 days without blood in stool and no danger signs.<br><br>**Definition of danger signs requiring referral**<br>General danger signs: Child is unable to drink, feed or breast feed; Vomits everything; Has or had convulsions.<br>Physical danger signs: Child has chest indrawing; Palmar pallor; Red on Mid-Upper Arm Circumference (MUAC) tape; Swelling of both feet (Bipedal oedema).
**Improving the quality of paediatric malaria diagnosis and treatment by rural providers in Myanmar: an evaluation of a training and support intervention**<br><br>Aung et al.<br><br>Malaria Journal (2015)	13 townships in central Myanmar<br><br>Repeated cross-sectional study	Malaria	Observation of management of illness in simulated 5-year-old patient (Observed Simulated Patient); score given out of 100	**Overall scores:**<br>Baseline (prior to training): 12/100<br>Midline (6 months after training): 48/100<br>Endline (12 months after training): 45/100<br><br>**Recognition of signs of severe malaria:**<br>Baseline (prior to training): 1.4/13<br>Midline (6 months after training): 2.9/13<br>Endline (12 months after training): 2.16/13<br><br>**Using RDT:**<br>Baseline (prior to training): 1.6/30<br>Midline (6 months after training): 22.92/30<br>Endline (12 months after training): 22.56/30<br><br>**Appropriate referral:**<br>Baseline (prior to training): 0.5/1<br>Midline (6 months after training): 0.87/1<br>Endline (12 months after training): 0.94/1<br><br>**Prescibing (treatment):**<br>Baseline (prior to training): 0.61/10<br>Midline (6 months after training): 4.72/10<br>Endline (12 months after training): 4.44/10	No information about CHW selection. The recruited cohort had to complete two 3-day trainings that was 1 month apart.	Diagnosing and treatment protocols were based on WHO protocols and PSI/Myanmar guidelines.

Title, Author, Journal, & Year	Country / Region and Study Design	Conditions Assessed	Assessment Method(s)	Correct Diagnosis / Treatment Proportions	CHW Selection & Training	Definition of Correct Diagnosis / Treatment
**Assessing the Quality of Care for Pneumonia in Integrated Community Case Management: A Cross-Sectional Mixed Methods Study**<br>Sinyangwe et al.<br>Plos One (2016)	Luapula province, Zambia<br>Cross-sectional mixed methods	Pneumonia	1497 CHW consultations directly observed, with measurement of respiratory rate for children with suspected pneumonia recorded by video - counts taken by CHWs compared against reference standard determined by experts from the video footage<br>3 focus group discussions and 9 in depth interviews to supplement	81% agreement between CHWs and experts for measuring respiratory rates<br>78% agreement between CHWs and experts for providing appropriate treatment	77% of CHWs had attained a secondary education. 90% worked in the same community where they originated from. All CHWs received a 6 day iCCM training. 79% of the CHWs had previously received a 6 week training from the Ministry of Health.	CHWs were trained in iCCM guidelines to identify suspected pneumonia: children who have fast breathing with cough or difficulty breathing. For the study to gain correct diagnosis of respiratory rates, CHWs decided whether or not to measure the respiratory rate for children with suspected pneumonia using a ARI timer. A respiratory rate of greater than/equal to 50 and greater than/equal to 40 indicates suspected pneumonia
**iCCM & community-based health planning and services: a cross sectional study on the effectiveness of the national implementation for the treatment of malaria, diarrhoea and pneumonia**<br>Ferrer et al.<br>Malaria Journal (2016)	Volta and Northern regions of Ghana<br>Repeated cross-sectional study	Malaria, diarrhea, pneumonia	Caretaker interview (household survey, asked mothers about child illnesses in past 2 weeks and any treatment by CBAs)	HBC:<br>Malaria: 23.8% of those with fever received ACT or were referred with artesunate to facility<br>Diarrhea: 9.2% of those with diarrhea received ORS or ORS + zinc, or were referred<br>Pneumonia: 26.9% of those with fast or difficult breathing got amoxicillin or were referred<br>CHPS:<br>Malaria: 45.5% tested for malaria; of those who tested positive, 22.2% were given ACT; for all uncomplicated malaria (those testing positive and those with fever and no lab confirmation, not referred), 14.8% received ACT<br>Diarrhea: 12.0% got ORS, 9.4% got zinc, and 0.9% got both<br>Pneumonia: 20.8% got amoxicillin or co-trimoxazole	HBC: CHW are the lowest based care. CHWs report their activities to care providers at the CHPS compounds. The CHWs were trained to identify and treat malaria, diarrhea, and pneumonia<br>CHPS: Care is provided by a resident community health nurse or community health officer	HBC<br>Malaria: all fever when no labs<br>Diarrhea: 3+ loose/watery stools in 24 hrs<br>Pneumonia: cough with fast or difficult breathing<br>CHPS<br>Malaria: all fever when no labs or malaria RDT was positive<br>Diarrhea: 3+ loose/watery stools in 24 hrs<br>Pneumonia: cough with fast or difficult breathing<br>See Table 1 for more definitions specific to treatment differentials
**Quality of care of treatment for uncomplicated severe acute malnutrition provided by lady health workers in Pakistan**<br>Rogers et al.<br>Public Health Nutrition (2017)	Sindh Province, Pakistan<br>Cross-sectional survey	Malnutrition	Direct observation only (with re-examination only for MUAC measurement)	Child correctly evaluated for danger signs: 65.0%<br>MUAC correctly measured: 57.4%	Each CHW had a minimum of a 8th grade standard formal education and received 2 years of training on family planning and basic health.	Quality of care was defined as the capacity of the CHW to evaluate, classify and treat cases of uncomplicated SAM, provide nutritional counseling, and refer complicated cases of SAM to inpatient care.

Title, Author, Journal, & Year	Country / Region and Study Design	Conditions Assessed	Assessment Method(s)	Correct Diagnosis / Treatment Proportions	CHW Selection & Training	Definition of Correct Diagnosis / Treatment
**Quality of care in iCCM services in Bugoye, Uganda: a retrospective observational study**<br>Miller et al.<br>Malaria Journal (2018)	Bugoye, Uganda<br>Retrospective observational	Malaria, pneumonia, diarrhea	Lot quality assurance sampling of individual patient records (register review)	97% of patients with diarrhea treated with ORS & zinc<br>95% of patients with pneumonia treated with amoxicillin<br>94% of patients with malaria treated with ACT or artesunate<br>44% of patients with negative RDT appropriately referred to facility	CHWs were volunteer workforce who had limited formal education. All CHWs were literate in the local language for the area that they were serving. They received 3 days of general training, 5 days of iCCM training, and half-day refresher trainings.	CHWs used the "Sick Child Job Aid" to help guide them through diagnosis and treatment for each patient. Disease specific quality metrics were as follows: (1) adherence to correct diagnosis protocol (defined as performing RDT for malaria in patients with a fever and measuring the respiratory rate for all patients with a cough/fast breathing), (2) appropriate prescribing practices, (3) appropriate referral processes with iCCM guidelines, (4) proportion of CHW providing high quality care using LQAS decision rule cut-off.
**Quality of Sick Child-Care Delivered by CHWs in Tanzania**<br>Baynes et al.<br>Int J Health Policy Manag (2018)	3 rural districts of Tanzania (intervention districts for Connect project)<br>Cross-sectional survey	Malaria, pneumonia, diarrhea	Direct observation and re-examination	Overall correct IMCI classification: 73%<br>Overall correct classification for only uncomplicated malaria, diarrhea, pneumonia: 83%<br>Uncomplicated malaria: 84%<br>Uncomplicated fever (no malaria): 84%<br>Uncomplicated cough / fast breathing: 74%<br>Uncomplicated diarrhea: 71%<br>Correct referral: 66%	CHWs were selected based on community requirements and the minimum eligibility criteria outline for civil service employment in Tanzania. These requirements include: “form-four” education level, residency in community that will be serving, and selection from their communities. CHWs underwent a 9 month training on CHW curriculum that incorporated the biological, clinical, and community aspects of primary health care.	CHWs were trained to assess, classify, and treat sick children in the home using an algorithm adapted from the IMCI approach.<br>Uncomplicated fever: Child with fever lasting less than 7 days and a negative response to m-RDT, but without any other danger signs. CHW treats with paracetamol.<br>Uncomplicated malaria: Child with fever lasting less than 7 days and a positive response to m-RDT, but without any other danger signs. CHW treats with ACT.<br>Uncomplicated cough with fast breathing: Child with cough for less than 21 days and with fast breathing (respiratory rate of 50 breaths per minute or more for ages 2-12 months and 40 breaths per minute for ages 12 months to 5 years). CHW treats with amoxicillin.<br>Uncomplicated diarrhea: Child with diarrhea for less than 14 days without blood in stool and no danger signs. CHW treats with ORS and zinc
**Using the RE-AIM framework to evaluate the implementation of iCCM in Kenya**<br>Onono et al.<br>Acta Paediatrica (2018)	Western Kenya<br>Prospective observational study	Diarrhea, pneumonia, malaria	Caregiver interviews (baseline and endline household surveys of mothers whose children had iCCM conditions in past 4 weeks), direct observation and re-examination (conducted through midline survey)	Caregiver interviews (baseline / endline):<br>% of children with diarrhea who got ORS & zinc: 9.1% / 58.3%<br>% of children with ARI symptoms who got antibiotics: 36.7% / 55.6%<br>% of children with fever who got ACT: 47% / 63.4%<br>(note: did not assess correct RDT usage)	CHWs were unpaid and trained through an 8-week training by health facility clinicians.	Children aged 2 to 59 months with diarrhea, fever and/or cough and/or difficult breathing identified during home visits by CHWs, or whose caregiver sought care from a CHW, were assessed, classified and treated. The CHW recorded the findings on a standardized sick child recording form adapted from WHO. Any child with danger signs of severe illness was referred as per the standard of care.

Title, Author, Journal, & Year	Country / Region and Study Design	Conditions Assessed	Assessment Method(s)	Correct Diagnosis / Treatment Proportions	CHW Selection & Training	Definition of Correct Diagnosis / Treatment
				Direct observation and re-examination proportion of cases correctly classified:<br>Pneumonia: 80.2%<br>Diarrhea: 80.2%<br>Malaria: 95.5%		
**Variation in the quality and out-of-pocket cost of treatment for childhood malaria, diarrhoea, and pneumonia: Community and facility based care in rural Uganda**<br>Soremekun et al.<br>Plos One (2018)	Mid-Western Uganda<br>Cross-sectional survey	Malaria, diarrhea, pneumonia	Caregiver interviews (household survey)	% of children with fever who received an RDT: 25%<br>% of children with suspected malaria (all children with fever who did not have a negative blood test) who were appropriately treated: 47%<br>% of children with confirmed malaria who were appropriately treated: 73%<br>% of children with diarrhea treated with ORS: 30%<br>% of children with diarrhea treated with ORS & zinc: 9%<br>% of children with pneumonia who were appropriately treated: 54%<br>Overall appropriate assessment / treatment: 47%	At the lowest level of care are the Village Health Team members (VHT), a voluntary cadre of CHW which are located in communities with otherwise poor access to health facilities	Case definitions of episodes of suspected malaria, diarrhea and suspected pneumonia, appropriate treatment for each of these diagnoses, care seeking and cost of care seeking were developed in accordance with standard WHO/UNICEF guidelines for treatment of malaria, diarrhoea and pneumonia in the community and at health facilities.
